# Molecular Rectangles Featuring Two Parallel *NCN*‐Coordinated Platinum Units: Enhancing Near‐Infrared Emission Through Excimer Formation

**DOI:** 10.1002/chem.202500834

**Published:** 2025-04-03

**Authors:** Rebecca J. Salthouse, Amit Sil, Piotr Pander, Fernando B. Dias, J. A. Gareth Williams

**Affiliations:** ^1^ Department of Chemistry Durham University South Road Durham DH1 3LE UK; ^2^ Faculty of Chemistry Silesian University of Technology Strzody 9 Gliwice 44‐100 Poland; ^3^ Centre for Organic and Nanohybrid Electronics Silesian University of Technology Konarskiego 22B Gliwice 44‐100 Poland; ^4^ Department of Physics Durham University Durham DH1 3LE UK

**Keywords:** excimer, luminescence, macrocycle, near‐infrared (NIR), platinum

## Abstract

New macrocycli**c molecules are** described that incorporate Pt(*NCN*) units on opposite edges of a rectangular structure, with xanthene units constituting the other two sides. Here, *NCN* represents a cyclometallating tridentate ligand based on 2,6‐di(2‐pyridyl)benzene or its pyrimidine analog. The complexes display strong photoluminescence peaking in the near‐infrared region of the spectrum in solution (*λ*_max_ up to 761 nm). Photophysical data and DFT calculations indicate that the emission arises from “intramolecular excimers”—triplet excited states that form when the two Pt(*NCN*) units within the molecule are brought into close proximity to interact interfacially. In doped polymers, the necessary molecular distortion is inhibited, but related excited states that emit in a similar region can still form through intermolecular interactions.

## Graphical abstract

Macro‐cycling into the near‐infrared: By incorporating two cyclometallated Pt(II) units into opposite sides of a molecular rectangle, the formation of near‐infrared‐emitting excited states that span the two units is promoted in solution, compared to the corresponding, acyclic, tweezer‐like complexes. A degree of molecular reorganization is nevertheless required to form such states, which is inhibited in more rigid media.

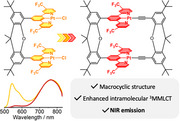

## 1 Introduction

Molecules that emit light efficiently in the deep‐red and near‐infrared (NIR) regions of the electromagnetic spectrum are sought after for technological and biomedical applications. For instance, there is increasing interest in NIR‐emitting organic light‐emitting diodes^[^ ^1^ ^]^ (NIR‐OLEDs) as alternatives to traditional ionic materials normally based on lanthanide‐doped oxides,^[^ ^2^ ^]^ in telecommunications.^[^ ^3^ ^]^ Meanwhile, molecules that absorb and emit at low energy are attractive in optical bioimaging and sensing, owing to the relative transparency of biological tissue in the 650–1400 nm range, and to the *λ* ^−4^ dependence of scattering.^[^ ^4^, ^5^, ^6^, ^7^, ^8^ ^]^ Nevertheless, the design of molecular materials that emit in the NIR region with high efficiency is challenging, since the transfer of energy from electronic excited states into vibrational states increases rapidly as the excited state energy decreases according to Fermi's golden rule, often referred to as the energy gap law.^[^ ^9^, ^10^, ^11^ ^]^ In the case of phosphors that rely on a heavy metal atom to promote emission from triplet states (by relaxation of the spin selection rule through spin‐orbit coupling), the problem is typically exacerbated by a decrease in metal character in the pertinent excited states when extended π‐conjugated ligands are used—the normal strategy for shifting to the red in molecular systems.^[^ ^12^, ^13^ ^]^

In that context, platinum(II) complexes differ from those of d^6^ metal ions like Ir(III) in that they are normally square‐planar. Face‐to‐face interactions between planar complexes may lead to bimolecular species that have lower‐energy excited states than the isolated molecules and which emit at lower energy.^[^ ^14^ ^]^ The behavior may be akin to classic fluorescent organic molecules like pyrene^[^ ^15^ ^]^—where the interaction occurs only in the excited state, forming excimers—and/or it may involve ground‐state interactions and the formation of dimers or higher aggregates.^[^ ^16^, ^17^, ^18^, ^19^, ^20^, ^21^, ^22^, ^23^, ^24^, ^25^, ^26^ ^]^ Either way, it offers an intriguing alternative approach for generating low‐energy emissions, of much interest in the design of deep‐red and NIR‐emitting OLEDs.^[^ ^1^, ^27^, ^28^, ^29^, ^30^ ^]^

**Figure 1:**
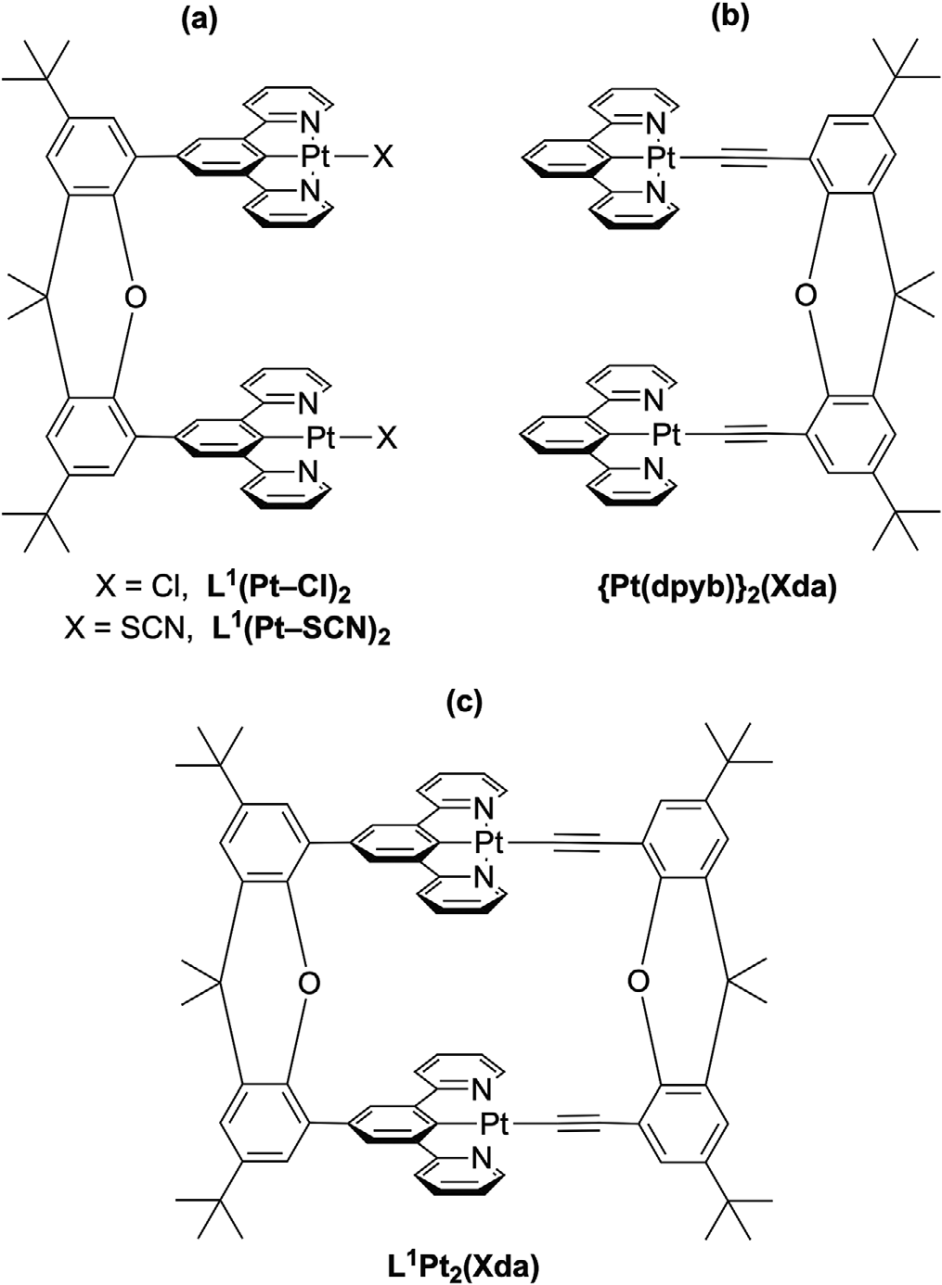
Examples of our previously reported tweezer‐like dinuclear complexes featuring Pt(dpyb) units connected to a xanthene linker, either (a) through the central phenyl ring,^[^ ^31^, ^32^ ^]^ or (b) through a monodentate acetylide ligand.^[^ ^32^ ^]^ The present work introduces a new class of macrocyclic dinuclear complex that features both types of connection, of which the parent example is shown in (c).

Nevertheless, both in solution and in a device, such bimolecular species inevitably require elevated concentrations of material to be formed in sufficient proportion for them to contribute significantly to the photo‐ or electroluminescence spectrum. Increasing the concentration is not necessarily viable in applications such as OLEDs, as it may adversely affect other properties such as charge transport. The covalent linkage of *two* Pt(II) units in such a way as to facilitate the requisite face‐to‐face interactions between them *intramolecularly*—as opposed to intermolecularly—can offer a way to circumvent this limitation.^[^ ^14^ ^]^ In earlier work we used a xanthene scaffold to connect two Pt(*NCN*) units, building upon the fact that mononuclear Pt(*NCN*)X complexes are often highly phosphorescent and form brightly‐emitting excimers [here, *NCN* represents a tridentate cyclometallating ligand based on 2,6‐dipyridylbenzene (dpybH), and *X* is a monodentate ligand such as a halide or acetylide].^[^ ^31^, ^32^, ^33^ ^]^ The connection between the Pt(II) units and the xanthene was made either directly to the central phenyl ring of the *NCN* ligand—as in L^1^(Pt─Cl)_2_^[^ ^31^, ^32^ ^]^ and L^1^(Pt─SCN)_2_^[^ ^33^ ^]^—or through a *para*‐substituted aryl acetylide acting as the monodentate ligand, as in {Pt(dpyb)}_2_(Xda)^[^ ^32^ ^]^ (Figure 1, H_2_Xda = 4,5‐bis‐ethynyl‐2,7‐di‐*tert*‐butyl‐9,9‐dimethyl‐xanthene or “xanthene diacetylene”). Both designs promote the formation of intramolecular interactions between the Pt(*NCN*) units, which leads to low‐energy emission (i.e., from excited states that span both units) predominating even at high dilution.

In the present work, we introduce a new class of dinuclear complexes, namely L^1^Pt_2_(Xda) and derivatives (Figure 1), that combines the two types of linkers in such a way as to generate a macrocyclic structure—a molecular rectangle in which two parallel edges incorporate Pt(*NCN*) units. We reasoned that such a structure might be expected not only to further facilitate the attainment of the low‐energy “Pt_2_” states on entropic grounds but also to reduce non‐radiative decay processes through the suppression of molecular motion, thus enhancing the efficiency of emission. The synthesis of these new molecular materials is described, together with their photophysical properties in solution and in polymer‐doped and neat films.

## 2 Results and Discussion

**Scheme 1:**
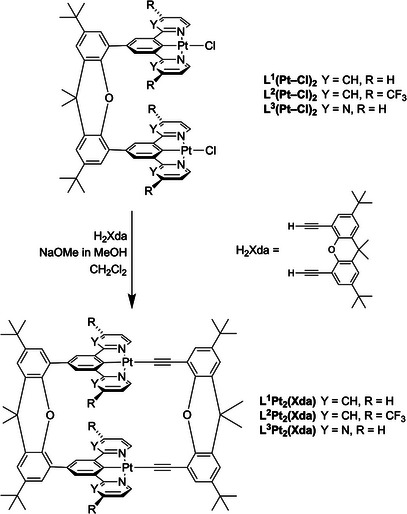
Synthetic route to the new macrocyclic complexes L^1–3^Pt_2_(Xda) studied in this work.

Three members of the new family of cyclic dinuclear complexes were prepared, as shown in Scheme 1. The metathesis of the chloride ligand in mononuclear Pt(*NCN*)Cl complexes by aryl acetylides is well established and takes place under mild conditions. The new dinuclear complex L^1^Pt_2_(Xda) was therefore synthesized using the same methodology by treating the chloro complex L^1^(Pt─Cl)_2_ with H_2_Xda in the presence of NaOMe as a base (experimental details are given in the Supporting Information). Apparently, the formation of the macrocyclic structure is favored over linear oligomerization, since the desired product was formed in high yield (> 80%). Related structures featuring Pt(II) terpyridyl acetylide units have previously been prepared by Yam and co‐workers,^[^ ^34^ ^]^ but they have a more open structure with a greater separation of the metallic units: the “vertical edges” of the rectangles comprise 2,6‐bis‐(3‐ethynylphenyl)pyridine units in that case, which are more flexible than the xanthene units here. The purpose of that work was to examine host‐guest inclusion within the resulting larger cavity, rather than promoting interactions between the metallic sides of the rectangle.

We also prepared two derivatives of the parent structure, namely L^2^Pt_2_(Xda), which carries trifluoromethyl substituents in the *para* positions of the pyridyl rings, and L^3^Pt_2_(Xda), the pyrimidine analog of the parent. The rationale for selecting these derivatives in the context of targeting NIR emission stems from our previous work on mononuclear complexes: we had found that the introduction of *p*‐CF_3_ substituents into the pyridine rings red‐shifts the excimer and the aggregate emission relative to Pt(dpyb)Cl,^[^ ^35^ ^]^ whilst the switch from pyridine rings to pyrimidines was accompanied by a red‐shift of the aggregate emission in the solid state.^[^ ^36^, ^37^ ^]^ These two dinuclear complexes were prepared in the same way as the parent L^1^Pt_2_(Xda), starting from L^2^(Pt─Cl)_2_ and L^3^(Pt─Cl)_2_, but they required more laborious purification by chromatography, which somewhat lowered the overall isolated yields (Scheme 1). The new materials were characterized by ^1^H and ^13^C NMR spectroscopy and high‐resolution mass spectrometry. Characterization data and spectra are given in the Supporting Information. The identities of L^1^Pt_2_(Xda) and L^2^Pt_2_(Xda) were further confirmed by x‐ray diffraction (Figures S8,9) but, unfortunately, both structures show extensive disorder in the packing of the molecules, such that geometric data cannot be reliably deduced.

**Figure 2:**
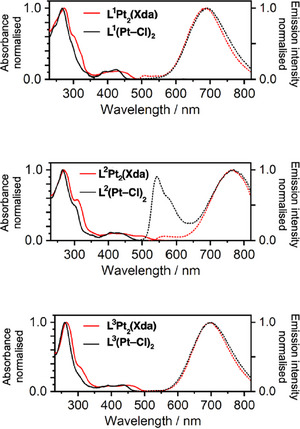
Absorption and photoluminescence spectra of L^1–3^Pt_2_(Xda) (solid and dotted red lines respectively), together with those of the corresponding parents L^1–3^(Pt─Cl)_2_ (solid and dotted black lines), in dilute CH_2_Cl_2_ solution at 295 K.

The absorption and photoluminescence spectra of the new macrocyclic complexes L^1–3^Pt_2_(Xda) in CH_2_Cl_2_ are shown in Figure 2 with those of the parent acyclic dinuclear complexes L^1–3^(Pt─Cl)_2_ for comparison; the photophysical data are summarized in Table 1. The absorption profiles of the macrocyclic structures broadly resemble those of the parents, with the pattern typical of Pt(dpyb)Cl—namely very intense bands < 330 nm due to π–π* transitions within the aromatic rings, accompanied by somewhat weaker bands around 400 nm attributed to charge‐transfer transitions involving the metal. However, inspection reveals that the lowest energy absorption bands of each cyclic complex L*^n^* Pt_2_(Xda) are red‐shifted and/or tail out significantly further to the red than the corresponding L*^n^* (Pt─Cl)_2_ parent. This effect is most likely due to interfacial interactions between the Pt(*NCN*) units within the molecule, giving rise to low‐energy excited states. Such interactions are frequently encountered in organoplatinum(II) complexes, as discussed in the introductory paragraphs, often leading to MMLCT states (metal–metal to ligand charge‐transfer) that are manifest by the emergence of low‐energy absorption bands.^[^ ^16^ ^]^

**Table 1:** Absorption and emission data of the cyclic complexes L*^n^* Pt_2_(Xda) (in boldface) together with the corresponding data for the acyclic precursors L*^n^* (Pt─Cl)_2_.

		Emission at 295 K^[^ ^b^ ^]^				Emission at 77 K^[^ ^c^ ^]^	
Complex	Absorption^[^ ^a^ ^]^ *λ*_max_ / nm	*λ*_max_ / nm short‐*λ* ^[^ ^d^ ^]^	*λ*_max_ / nm long‐*λ* ^[^ ^d^ ^]^	*Φ*_lum_^[^ ^e^ ^]^	*τ* / ns^[^ ^f^ ^]^	*λ*_max_ / nm	*τ* / ns^[^ ^f^ ^]^
**L^1^Pt_2_(Xda)**	**273, 303sh, 406sh, 431**	–	**0.81**	**0.30**	**930**	**506, 541, 583**	**6500**
L^1^(Pt–Cl)_2_^[^ ^g^ ^]^	272, 371, 391, 425	–	0.77	0.25	1500	501, 537, 578	12000
**L^2^Pt_2_(Xda)**	**270, 308, 415, 433, 494**	–	**761**	**0.11**	**420**	**550, 596, 639sh**	**5400**
L^2^(Pt–Cl)_2_^[^ ^g^ ^]^	270, 304, 357, 380, 412, 425	542, 582	766	0.12	7000 at 542 nm 450 at 766 nm	540, 580	9400
**L^3^Pt_2_(Xda)**	**265, 308sh, 396, 449**	–	**697**	**0.18**	**720**	**506, 528, 571**	**5400**
L^3^(Pt–Cl)_2_	264, 374sh, 390, 435	–	698	0.16	760	500sh, 517	4100

^[a]^ In CH_2_Cl_2_ at 295 K. Molar absorptivities *ε* are given in the Supporting Information.

^[b]^ In degassed CH_2_Cl_2_ at 295 K.

^[c]^ In diethyl ether/isopentane/ethanol, 2:2:1, v/v.

^[d]^ L^2^(Pt─Cl)_2_ displays two sets of bands of comparable intensity in dilute solution (see Figure 2), one at higher energy (“short‐λ” column) and one at lower‐energy (“long‐λ” column), whereas for the other complexes, the intensity of the former band is essentially negligible.

^[e]^ Photoluminescence quantum yields were measured using [Ru(bpy)_3_]Cl_2(aq)_ as the standard.

^[f]^ Lifetimes measured by time‐correlated single‐photon counting following excitation at 405 nm.

^[g]^ Data from reference [32].

All three complexes are brightly photoluminescent in deoxygenated solution at ambient temperature, displaying essentially only one broad band in the deep‐red / NIR region (Figure 2). These bands resemble the excimer bands displayed by the mononuclear complexes like Pt(dpyb)Cl at elevated concentration,^[^ ^38^ ^]^ and the excitation spectra closely match the absorption spectra (Figure S10). The trend in the emission *λ*_max_ values (690, 761, and 697 nm for the complexes of L^1^, L^2^, and L^3^ respectively) is consistent with that displayed by the excimers of the corresponding mononuclear PtL*^n^* Cl complexes and discussed elsewhere.^[^ ^36^, ^37^ ^]^ However, the behavior differs from the mononuclear complexes in that (i) the emission spectra and lifetimes are independent of concentration, and (ii) there is no evidence of a rise‐time for the emission on the timescale investigated (Figure S11; *cf*. the excimer emission of Pt(dpyb)Cl, which has a rise‐time of the order of hundreds on nanoseconds^[^ ^39^ ^]^). These observations point to an *intramolecular* origin of the excited states spanning the two Pt(NCN) units. For L^1^Pt_2_(Xda) and L^2^Pt_2_(Xda), there is additionally a hint of very weak, higher‐energy emission (500–550 nm and 550–600 nm, respectively). Based on the behavior of the acyclic {Pt(dpyb)_2_}(Xda) analogs (which showed significant contributions in both the green and red regions), this emission is attributed to excited states on non‐interacting Pt(*NCN*) units.

Further insight on this point is provided by comparison with the corresponding acyclic L^1–3^(Pt─Cl)_2_ complexes. For the complexes of L^2^, there is a striking difference between the spectra of the cyclic versus the acyclic parent. Thus, L^2^(Pt─Cl)_2_ showed *two* emission bands of roughly equal intensity—corresponding to the excited states associated with a single Pt(*NCN*) unit at higher energy and those spanning two such units at lower energy—while in L^2^Pt_2_(Xda), the former band has almost negligible intensity. It appears, then, that either (i) the formation of the latter excited states through the intramolecular interaction of Pt(*NCN*) units is indeed favored by cyclization, or (ii) non‐radiative decay of these states is suppressed in the macrocycle leading to proportionally higher intensity of the low‐energy, or (iii) most likely—a combination of both of these effects. For L^1^Pt_2_(Xda) and L^3^Pt_2_(Xda), the spectra are essentially the same as those of L^1^(Pt─Cl)_2_ and L^3^(Pt─Cl)_2_ respectively (which already showed uniquely the low‐energy band). The quantum yields are slightly increased upon the formation of the macrocycles,^‡^ likely due to the greater rigidity of the macrocyclic system compared to the acyclic analog, with less conformational freedom serving to suppress the non‐radiative deactivation.^[^ ^40^ ^]^

**Figure 3:**
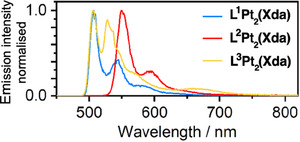
Photoluminescence spectra of L^1–3^Pt_2_(Xda) at 77 K in a glass of diethyl ether/isopentane/ethanol (2:2:1 v/v).

In a dilute glass at 77 K, the emission spectra show only one set of vibrationally structured bands at higher energy, in the region expected for emission from excited states confined to a single Pt(*NCN*) unit (Figures 3 and S12). There is no low‐energy emission. This finding is consistent both with experimental observations and DFT calculations on L^1–3^(Pt─Cl)_2_, where we concluded that significant distortion of the xanthene was required to bring the Pt(*NCN*) units sufficiently close together to form the excimer, a process that is evidently thermally activated.^[^ ^32^ ^]^

**Figure 4:**
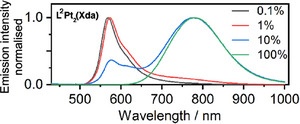
Photoluminescence spectra of L^2^Pt_2_(Xda) doped into polystyrene films at the concentrations indicated. Corresponding spectra for L^1^Pt_2_(Xda) and L^3^Pt_2_(Xda) are shown in the Supporting Information, Figure S17.

The behavior in the solid state also differs from the solution. The emission spectra of the complexes doped into thin films of polystyrene at low concentrations (0.1% by weight) show only the high‐energy emission band characteristic of excited states localized on a single Pt(*NCN*) unit (Figures 4 and S13). This behavior resembles that at 77 K and can be accounted for similarly: the more rigid environment of the solid state compared to the solution inhibits the attainment of the conformation required to form the low‐energy‐emitting states. Although the idealized structures of Figure 1 might suggest a Pt···Pt separation comparable to the C···C separation of the xanthene carbons, the xanthene unit offers a degree of flexibility that accommodates bowing of the structure, as evident, for example, from the crystal structures of some of the acyclic analogs, where Pt···Pt separations were in excess of 4.5 Å, too long to allow intramolecular excimeric states to form. The deficiencies in the attempted diffraction studies in the present work do not allow us to determine the separation reliably. Nevertheless, this flexibility of the xanthene unit has been observed elsewhere; e.g., in a xanthene appended with two Pd porphyrins, the Pd···Pd separation is around 4 Å.^[^ ^41^ ^]^ Meanwhile, in other systems, wherein intermetallic interactions are particularly favored, planar groups attached to the xanthene bridge may be bowed *inwards*; e.g., Chan et al. found an intramolecular Pt···Pt separation as short as 3.22 Å for two such Pt‐salphen units.^[^ ^42^ ^]^

Time‐resolved measurements confirm the differing origins of the bands. The higher‐energy‐emitting excited state has a longer lifetime that does not change significantly with concentration, indicating that the lower‐energy‐emitting excited state is not formed from it (as would be the case for an excimer). The lower‐energy (aggregate) band decays more rapidly. Pertinent decay traces are shown in Figures S15–17 and the data are summarized in Table S2. Interestingly, powdered samples of L^1^Pt_2_(Xda) and L^3^Pt_2_(Xda)—as isolated from the original synthesis and purification—show different proportions of the two bands compared to the neat films obtained by evaporation, with a proportionately much lower contribution of the lower‐energy band (Figure S14). This no doubt reflects the subtle but important influence of the solvent, temperature, and evaporation rate on the packing of the molecules in the solid state, as previously encountered for some mononuclear Pt(*NCN*)Cl complexes.^[^ ^43^ ^]^ Conversely, L^2^Pt_2_(Xda) in the powder shows only the low‐energy band, just as in the neat film. Presumably, in this instance, the same (or similar) arrangement of molecules is attained through bulk evaporation of CH_2_Cl_2_ and MeOH after chromatography to that present in the films.

Support for the need for conformational rearrangement of the molecule to give the low‐energy emission band is provided by density functional theory (DFT) and time‐dependent DFT (TD‐DFT) calculations (details of the methods are given in Supporting Information). The discussion here will be confined to the parent L^1^Pt_2_(Xda), but comparable results on the other systems have also been obtained (discussed in the Supporting Information). The optimized geometries of the ground S_0_ and excited T_1_ states of L^1^Pt_2_(Xda) are found to be markedly different, with Pt···Pt distances of 4.47 and 2.93 Å respectively. The latter is clearly short enough to account for the observed low‐energy emission in solution from a state spanning both of the Pt(*NCN*) units. Evidently, the molecule must undergo a profound structural reorganization to attain this T_1_ geometry, and the barrier to such a rearrangement in the solid or glass at 77 K accounts for the contrasting experimental results under those conditions. The structural reorganization involves a substantial distortion of the xanthene unit embedded within the L^1^ ligand (with an angle of 141° between the constituent phenyl rings), but not of the xanthene of Xda. In the ground state, L^1^Pt_2_(Xda) displays two degenerate singlet and triplet excitations of MLCT nature, localized on the Pt(*NCN*)X units, much as for L^1^(Pt─Cl)_2_ (see Figures S18 and S20, and Tables S3–S5). The T_1_ geometry represents a typical orbital pairing for the S_1_ and T_1_ excited states with intramolecular MMLCT character (Figures S19 and S21). Similar results are found for the complexes of L^2^ and L^3^ (Figures S22 to S25).

The above outcomes are in line with conclusions from our earlier calculations on the acyclic complexes of type (a) and (b) in Figure 1. They showed that those of type (a) require a more substantial distortion of the xanthene unit to arrive at the MMLCT‐type excited state—with a short Pt···Pt distance—than those of type (b), where there is more flexibility through the acetylide linkers. Thus, the cyclic complexes have behavior akin to the type (a) acyclic parents, displaying strong intramolecular ^3^MMLCT emission only in solution. Conversely, the restriction of molecular motion in the solid state or in a frozen glass leads to purely ^3^MLCT emission associated with a single Pt(*NCN*) unit, from species that have a ground state‐like geometry. When ^3^MMLCT emission appears in the solid state, it is due to *intermolecular* interactions.

## 3 Conclusion

In conclusion, the strategy of incorporating two Pt(*NCN*) units within a cyclic structure is found to offer efficient phosphorescence in the NIR region in solution. The cyclic design favors low‐energy emission in solution from excited states spanning two Pt(*NCN*) units of a type that, in mononuclear analogs, requires high concentrations to form. Moreover, it favors the formation and emission from such states compared to acyclic analogs, as seen most strikingly in the case of L^2^Pt_2_(Xda), which shows uniquely low‐energy emission in solution, in contrast to the dual emission of L^2^(Pt─Cl)_2_. That is particularly significant because it is this more electron‐deficient L ligand that gives the lowest‐energy emitting excimeric states. The attainment of such states still requires significant structural reorganization, which is inhibited in rigid media (solids, doped films, and frozen glasses). Nevertheless, in films (relevant to OLEDs), the emission from low‐energy excited states formed *intermolecularly* is also favored by the cyclic structure. Thus, whilst L^2^(Pt─Cl)_2_ shows roughly equal intensities of the two bands, neat films of L^2^Pt_2_(Xda) display the low‐energy emission uniquely, paving the way to NIR‐OLEDs using molecules based on this design. Further modifications to the structure can be envisaged that will help to impose the necessary mutual disposition of the Pt(*NCN*) units required for low‐energy emission. For example, an additional covalent linkage of one (or both) of the pyridine rings on the individual Pt(NCN) units could help to inhibit the bowing or twisting of the planes relative to one another, facilitating intramolecular interactions. The preparation of such structures would, however, be challenging. Meanwhile, removal of the sterically encumbering *t*‐butyl groups on one or both xanthene units may facilitate intermolecular interactions, though this would likely come at the expense of solubility. Finally, we note that the cavity size may be tuned to explore the macrocyclic structure as a host for suitable guest molecules, as has been explored in related systems.^[^ ^34^, ^44^, ^45^ ^]^

## Supporting Information

The authors provide more details of the following in the Supporting Information: experimental methods; synthetic details and characterization of new materials; crystallography; additional absorption and emission data; results of DFT and TD DFT calculations.

Institute and/or researcher Twitter usernames: @DurhamChemistry @Rebecca_Salty @aamitsil

## Conflict of Interests

There are no conflict of interest to declare.

## Supporting information



Supporting Information
