# Supplementary material for: Molecular Rectangles Featuring Two Parallel NCN‐Coordinated Platinum Units: Enhancing Near‐Infrared Emission Through Excimer Formation
Source: Chemistry. 2025 Apr 3;31(26):e202500834. doi: 10.1002/chem.202500834 (PMC12063040; doi:10.1002/chem.202500834)
Supplement: Supplementary file 1 — Supporting Information [file CHEM-31-e202500834-s001.pdf]

Supporting Information

Molecular Rectangles Featuring Two Parallel *NCN*-Coordinated Platinum Units: Enhancing Near-infrared Emission through Excimer Formation

Rebecca J. Salthouse,[a] Amit Sil,[a] Piotr Pander,[b,c,d] Fernando B. Dias[d] and J. A. Gareth Williams[a*]

[a]*Department of Chemistry, Durham University, Durham, DH1 3LE, U.K.*
** E-mail: j.a.g.williams@durham.ac.uk*
[b] *Faculty of Chemistry, Silesian University of Technology, Strzody 9, 44-100 Gliwice, Poland.*
[c] *Centre for Organic and Nanohybrid Electronics, Silesian University of Technology, Konarskiego 22B, 44-100 Gliwice, Poland.*
[d] *Department of Physics, Durham University, South Road, Durham, DH1 3LE, U.K.*

Section 1 Experimental methods and procedures 2
Materials and instrumentation for synthesis 2
Solution-state photophysics 2
Solid-state photophysics 2
Calculations 3
Section 2 Synthetic details 4
Intermediates and Precursors 4
Macrocyclic complexes 7
^{1}H NMR Spectra 12
Section 3 Crystallography 15
Section 4 Solution-state photophysics 17
Section 5 Solid-state photophysics 19
Section 6 Calculations 23
References 27

Section 1 Experimental methods and procedures

Materials and instrumentation for synthesis

Commercial chemicals were used as supplied without further purification. Reactions requiring an inert atmosphere were carried out using Schlenk-line techniques under an atmosphere of argon or nitrogen. Thin layer chromatography analysis was performed on F254 silica plates and visualized by UV irradiation at 254 and 365 nm. NMR spectra were recorded on a Varian VNMRS-600 or -700 spectrometer at the frequencies indicated. Two-dimensional NMR experiments, including homonuclear correlation spectroscopy (COSY), heteronuclear multiple bond correlation spectroscopy (HMBC) and heteronuclear single quantum coherence spectroscopy (HSQC), were used to facilitate the assignment of signals. Chemical shift values (δ) are reported in parts per million (ppm), coupling constants (J) are reported in Hz, and the spectra are referenced to residual solvent peaks: $CDCl_3$ = 7.26 and 77.2 ppm (^1H and ^{13}C respectively); CD_2Cl_2 = 5.32 and 53.8 ppm. Electrospray ionisation mass spectra (ESI) were recorded using a Waters Acquity TQD Tandem Quadrupole mass spectrometer with either acetonitrile or methanol as the carrier solvent. Atmospheric pressure solids analysis probe (ASAP) ionization mass spectra were obtained using a LCT Premier XE mass spectrometer and an Acquity UPLC from Waters Ltd at 350°C. High-resolution mass spectra were obtained using a Quantum time-of-flight (QTof) mass spectrometer.

Solution-state photophysics

UV-visible absorption spectra were recorded on a Biotech instruments Uvikon XS Spectrometer. Samples were contained in optically matched quartz cuvettes with a path length of 1 cm. Emission and excitation spectra in solution were acquired using a Horiba Fluoromax-2 spectrometer equipped with a Hamamatsu R928 photomultiplier tube. Samples were contained with quartz cuvettes modified to allow connection to a vacuum line. The solutions were degassed prior to measurements by three freeze-pump-thaw cycles, to a base pressure $< 10^{-2}$ mbar. Emission spectra were corrected for the spectral response of the detector. Excited-state lifetime measurements were made using an Edinburgh Instruments OB 920 fluorimeter with a pulsed laser diode operating at 405 nm. Spectra at 77 K were obtained in 4 mm o.d. tubes held within a glass Dewar.

Solid-state photophysics

The emission spectra of the films were recorded using a QePro compact spectrometer (Ocean Optics). Time-resolved measurements were undertaken with a Horiba DeltaFlex system using a 330 nm SpectraLED or a 405 nm Delta Diode light source. Temperature-dependent experiments in films were

conducted using a liquid nitrogen cryostat VNF-100 (sample in flowing vapour, Janis Research) under nitrogen atmosphere, while measurements at room temperature were recorded under vacuum in the same cryostat.

Calculations

DFT / TD-DFT calculations were performed using Orca 5.0.3 software[1–3] and the results were visualised using Gabedit 2.5.0.[4] Ground state (S_0) and triplet excited state (T_1) geometry were calculated using the BP86[5] functional and the def2-svp[6] basis set for all atoms with an effective core potential (ECP) for platinum. Single-point energy calculations were conducted by TD-DFT with Tamm-Dancoff approximation (TDA), using CAM-B3LYP[7] functional and the same basis set. Calculation time was greatly reduced with the RIJCOSX[8,9] approximation applied as a default setting with def2/J[10] auxiliary Coulomb-fitting basis set, and tight SCF and geometry optimization criteria. The calculation uses atom-pairwise dispersion correction with the Becke-Johnson damping scheme (D3BJ)[11,12] for both geometry optimisations and single-point energy calculations. All MO iso surfaces were rendered with iso value of 0.03 if not stated otherwise. All geometry optimisations were followed by vibrational frequency calculations to confirm genuine minimal energy configurations.

Section 2 Synthetic details

Intermediates and Precursors

The acyclic precursors $L^1(Pt–Cl)_2$ and $L^2(Pt–Cl)_2$ were prepared as described in our previous work.[14] The pyrimidine analogue $L^3(Pt–Cl)_2$ was prepared using a similar procedure, which is summarized in Scheme S1 below, followed by the synthetic details and characterization for each of the intermediates leading to it.

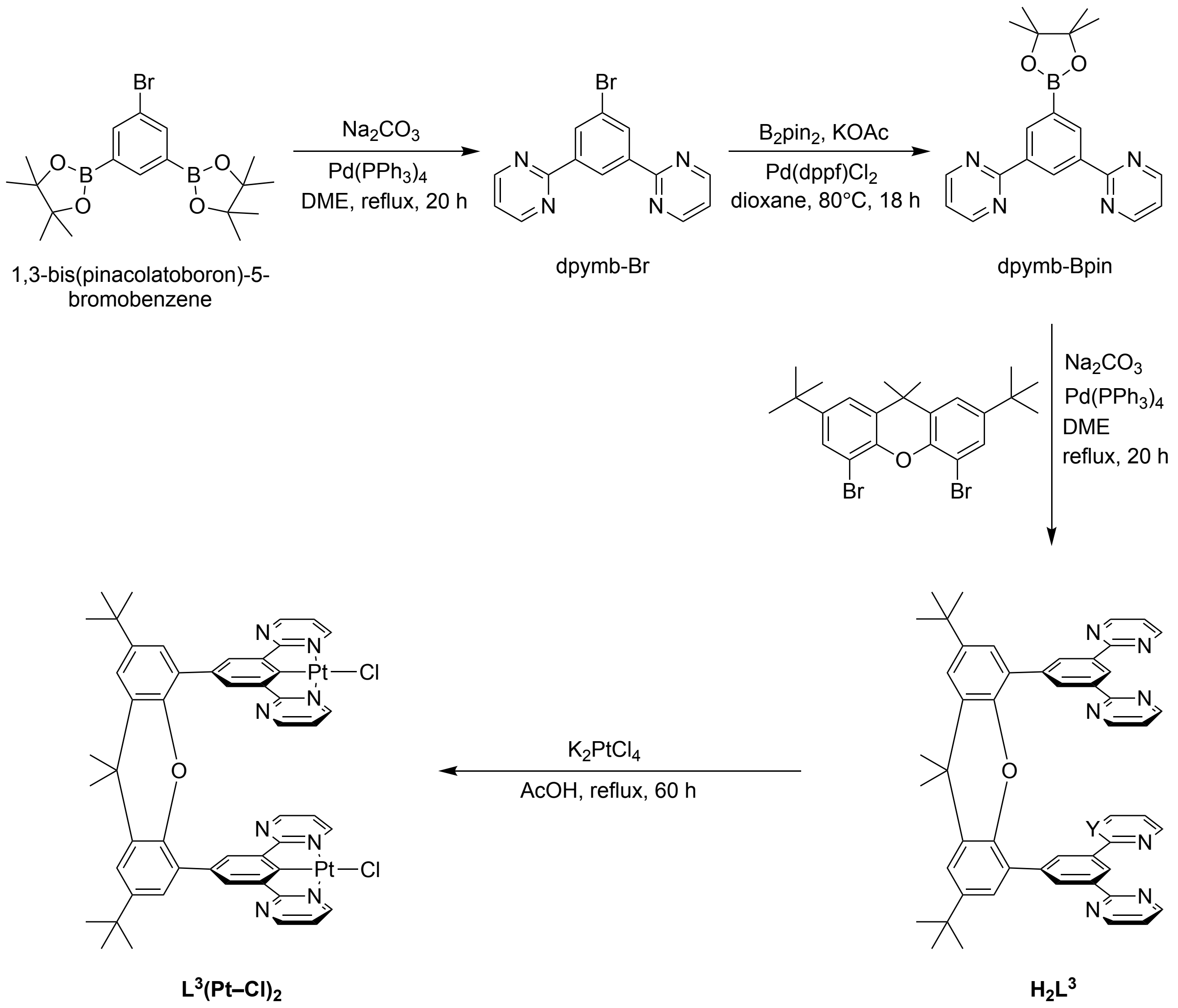

Scheme S1 The synthesis of $L^3(Pt–Cl)_2$ following the method established previously for $L^1(Pt–Cl)_2$.

Dpymb-Br

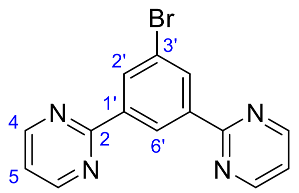

This compound was prepared by Suzuki cross-coupling of 1,3-di(pinacolatoboron)-5-bromobenzene (2.59 g, 6.35 mmol, prepared as described previously[14]) with 2-bromopyrimidine (2.02 g, 12.7 mmol). The two reagents were added to a Schlenk flask with aqueous Na_2CO_3 (1 M, 5.39 g, 50.8 mmol) and dimethoxyethane (40 mL). The mixture was degassed using three freeze-pump-thaw cycles, and $Pd(PPh_3)_4$ (0.367 g, 0.318 mmol) was then added under a flow of nitrogen. The mixture was heated at reflux for 20 h, then washed with water and extracted into dichloromethane (DCM). The solution was dried over anhydrous $MgSO_4$, filtered, and the solvent removed under reduced pressure. Purification was carried out by column chromatography on silica (hexane : ethyl acetate gradient, R_f = 0.3 in 70:30) to yield the title compound as a white solid (0.717 g, 36%). ^1H NMR (600 MHz, $CDCl_3$) δ_H = 9.48 (1 H, q, J 1.7, $H^{6'}$), 8.87 – 8.80 (4 H, m, H^4), 8.75 – 8.71 (2 H, m, $H^{2'}$), 7.24 (2 H, d, J 7.3, H^5). ^{13}C NMR (151 MHz, $CDCl_3$) δ_C = 163.1 (C^2), 157.3 (C^4), 139.8 ($C^{1'}$), 133.2 ($C^{2'}$), 126.6 ($C^{6'}$), 123.3 ($C^{3'}$), 119.7 (C^5). HRMS (ES^+) *m/z* = 313.0103 $[M+H]^+$, calc. for $[C_{14}H_{10}N_4Br]$ 313.0089.

Dpymb-Bpin

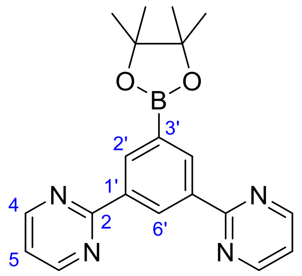

This compound was prepared by reaction of dpymb-Br (375 mg, 0.194 mmol) with B_2pin_2 (365 mg, 1.43 mmol) in the presence of KOAc (703 mg, 7.16 mmol) and $Pd(dppf)Cl_2$ (87 mg, 0.119 mmol), in 1,4-dioxane (10 mL), using the same procedure as that used for the pyridine analogue described previously.[14] The crude product was purified by column chromatography on silica (hexane : ethyl acetate gradient, 60:40) to yield the desired boronate ester as a pale yellow solid (350 mg, 81%). ^1H NMR (600 MHz, $CDCl_3$) δ_H = 9.64 (1 H, t, J 1.8), 9.02 (2 H, d, J 1.9), 8.88 (4 H, d, J 4.8), 7.25 (2 H, d, J 7.7), 1.38 (12 H, s). HRMS (ES^+) *m/z* = 361.395 $[M+H]^+$, calc. for $[C_{20}H_{22}N_4BrO_2]$ 361.184.

H_2L^3

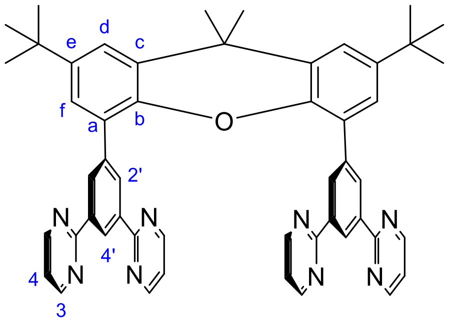

This compound was prepared by Suzuki cross-coupling of dpymb-Bpin (598 mg, 1.66 mmol) and 4,5-dibromo-2,7-di-*tert*-butyl-9,9-dimethylxanthene (362 mg, 0.755 mmol), in dimethoxyethane (6 mL) in the presence of aqueous Na_2CO_3 (640 mg, 6.04 mmol) and $Pd(PPh_3)_4$ (44 mg, 0.038 mmol). The procedure was as for dpymbBr above. The crude mixture was purified by column chromatography on silica (hexane : ethyl acetate gradient, $R_f = 0.1$ in 20:80) to yield H_2L^3 as an off-white solid (356 mg, 60%). ^{1}H NMR (600 MHz, $CDCl_3$) $\delta_H =$ 8.92 (2 H, d, J 1.8, $H^{6'}$), 8.54 (8 H, d, J 4.4, H^3), 8.46 (4 H, d, J 1.4, $H^{2'}$), 7.45 (2 H, d, J 2.3, H^d), 7.26 (2 H, d, J 2.3, H^f), 6.72 (4 H, t, J 4.5, H^4), 1.81 (6 H, s, H^{Me}), 1.36 (18 H, s, H^{tBu}). ^{13}C NMR (151 MHz, $CDCl_3$) $\delta_c = 163.9$ (C^1), 156.6 (C^3), 144.9 (C^e), 144.8 (C^b), 138.7 (C^c), 136.6 ($C^{3'}$), 131.7 ($C^{2'}$), 128.9 ($C^{1'}$), 128.8 (C^a), 126.0 ($C^{4'}$), 125.7 (C^f), 122.4 (C^d), 118.1 (C^4), 34.7 (C^g), 34.5 (C^h), 33.5 (C^{Me}), 31.6 (C^{tBu}). MS ESI (ES^+) *m/z* = 787.4 ($[M+H]^+$, 100%); HRMS (ES^+) *m/z* 787.3875 $[M+H]^+$, calc. for $[C_{51}H_{47}N_8O]$ 787.3873.

$L^3(Pt–Cl)_2$ – formed with its mononuclear analogue $HL^3(Pt–Cl)$

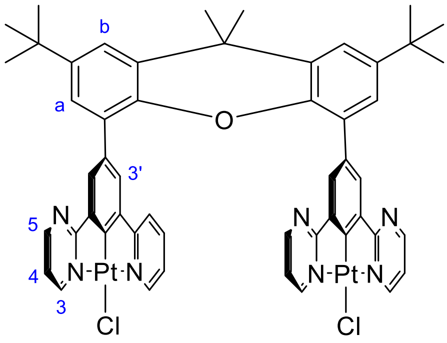

$L^3(Pt–Cl)_2$

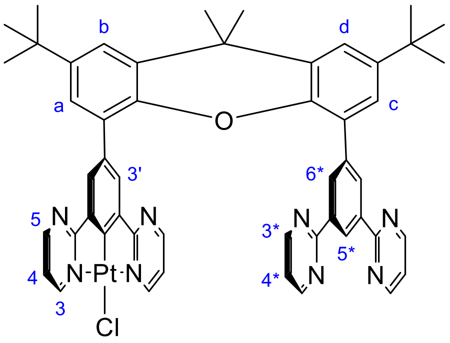

$HL^3(Pt–Cl)$

Potassium tetrachloroplatinate(II) (161 mg, 0.388 mmol) was added to a solution of H_2L^3 (61 mg, 0.078 mmol) in acetic acid (6 mL) in a Schlenk flask and the solution was degassed using three freeze-pump-thaw cycles. The reaction mixture was then heated at reflux for 60 h under nitrogen. The crude solid that precipitated upon cooling was purified by preparative column chromatography on silica (DCM : MeOH gradient) to yield a mixture of the desired dinuclear product $L^3(Pt–Cl)_2$ as an orange solid (10 mg, 10%) and the mononuclear HL^3PtCl as a yellow solid (22 mg, 28%).

L^3(Pt–Cl)$_2$: ^{1}H NMR (600 MHz, $CDCl_3$) δ_H = 9.45 (4 H, dd, J 5.7, 2.3, H^3), 8.47 (4 H, dd, J 4.7, 2.3, H^5), 7.78 (4 H, s, $H^{3'}$), 7.48 (2 H, d, J 2.4, H^b), 7.24 (2 H, d, J 2.3, H^a), 7.10 – 7.04 (4 H, m, H^4), 1.79 (6 H, s, H^{Me}), 1.38 (18 H, s, $H^{t\text{-Bu}}$). MS ASAP (AP^+) *m/z* 1209.3 [M-Cl]$^+$; HRMS (AP^+) *m/z* 1208.3348 [M-Cl]$^+$, calc. for [$C_{51}H_{44}ClN_8O^{194}Pt^{195}Pt$] 1208.2660.

HL^3(Pt–Cl): ^{1}H NMR (600 MHz, $CDCl_3$) δ_H = 9.29 (2 H, dd, J 5.7 2.3, H^3), 8.88 (1 H, t, J 1.7, H^{5*}), 8.62 (4 H, d, J 4.7, H^{3*}), 8.53 (2 H, d, J 4.7, H^{6*}), 8.44 (2 H, dd, J 4.7, 2.2, H^5), 7.75 (2 H, s, $H^{3'}$), 7.48 (1 H, d, J 2.3, H^b), 7.46 (1 H, d, J 2.4, H^d), 7.27 (2 H, d, J 2.3, H^a and H^c), 6.95 (2 H, dd, J 5.7, 4.7, H^4), 6.81 (2 H, t, J 4.7, H^{4*}), 1.80 (6 H, s, H^{Me}), 1.39 (9 H, s, $H^{t\text{-Bu}}$), 1.37 (9 H, s, $H^{t\text{-Bu}}$). ^{13}C NMR (151 MHz, $CDCl_3$) δ_c = 157.9 (C^3), 157.8 (C^5), 156.9 (C^{3*}), 131.9 (C^{6*}), 131.3 ($C^{3'}$), 126.0 (C^a), 125.6 (C^{5*}), 125.3 (C^c), 118.3 (C^{4*}), 117.8 (C^4), 33.5 (C^{Me}), 31.6 ($C^{t\text{-Bu}}$). MS ASAP (AP^+) *m/z* 1017.3 [M+H]$^+$; HRMS (AP^+) *m/z* 1017.3191 [M+H$^+$], calc. for [$C_{51}H_{45}ClN_8OPt$] 1017.3137.

Macrocyclic complexes

L^1Pt_2(Xda)

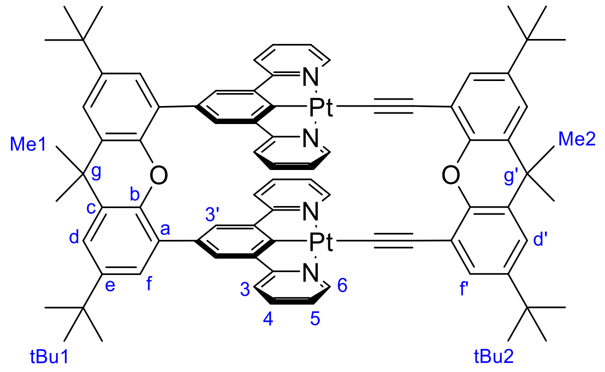

A mixture of 2,7-di-*tert*-butyl-4,5-diethynyl-9,9-dimethylxanthene (H_2Xda, 9 mg, 0.024 mmol) and NaOMe (4 mg, 0.73 mmol) in dry MeOH (20 mL) was stirred at room temperature for 30 min. A solution of L^1(Pt−Cl)$_2$ (30 mg, 0.024 mmol) in dry DCM (20 mL) was added and the resulting mixture stirred at 60°C for 48 h under an atmosphere of nitrogen. The solvent was then removed under reduced pressure and the resulting solid washed with water, MeOH and Et_2O. Recrystallisation from DCM / hexane gave the desired product as a yellow solid (31 mg, 83%). ^{1}H NMR (700 MHz, $CDCl_3$) δ_H = 9.72 – 9.63 (4 H, m, H^6), 7.56 (2 H, d, J 2.4, H^d or H^f), 7.48 (2 H, d, J 2.3, H^d or H^f), 7.37 (4 H, s, $H^{3'}$), 7.28 – 7.22 (8 H, m, H^4, H^d and H^f), 7.17 (4 H, d, J 7.8, H^3), 6.96 – 6.90 (4 H, m, H^5), 1.82 (6 H, s, H^{Me}), 1.70 (6 H, s, H^{Me}), 1.39 (18 H, s, H^{tBu}), 1.35 (18 H, s, H^{tBu}). ^{13}C NMR (176 MHz, $CDCl_3$) δ_c = 179.1, 168.9, 156.6 (C^6), 148.7, 145.5 ($C^{e/e'}$), 144.4, 143.9 ($C^{e'/e}$), 142.5, 141.7, 136.7 ($C^{4/d'/f'}$), 132.4, 129.9, 129.1 ($C^{c/c'}$), 129.1 ($C^{d/f}$), 128.8 ($C^{c/c'}$), 125.7 ($C^{4/d'/f'}$), 124.9 ($C^{3'}$), 123.0 (C^5), 122.2 ($C^{d/f}$), 120.0 ($C^{4/d'/f'}$), 118.3 (C^3), 116.0, 53.4, 50.9, 34.8, 34.6 ($C^{g/g'}$), 34.4 ($C^{g/g'}$), 33.6 (C^{Me}), 33.1

(C^{Me}), 31.6 (C^{tBu}), 31.6 (C^{tBu}), 31.4. MS ASAP (AP^+) *m/z* 1540.2 $[M+H]^+$; HRMS (AP^+) *m/z* 1540.5468 $[M+H]^+$, calc. for $[C_{82}H_{76}N_4O_2Pt_2]$ 1540.5363; Anal. calc. for $C_{82}H_{76}N_4O_2Pt_2{\cdot}3CH_2Cl_2$: C, 56.89; H, 4.61; N, 3.12 %; found C, 56.63; H, 4.96; N, 2.98 %. λ_{max} / nm (ε / M^{-1} cm^{-1}): 273 (78000), 303sh (44300), 406sh (8540), 431 (9020).

L^2Pt_2(Xda)

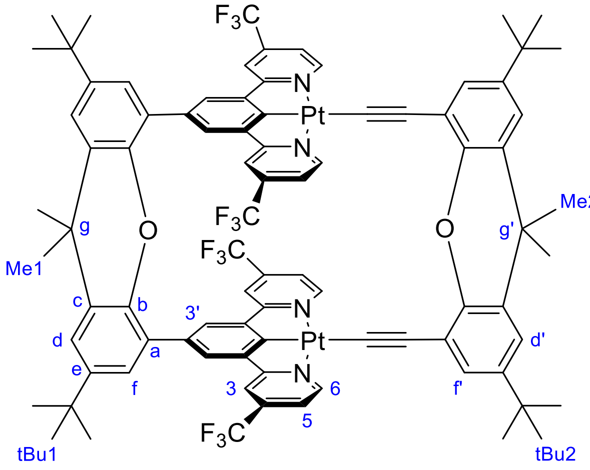

This compound was prepared similarly, from H_2Xda (5.6 mg, 0.015 mmol) and NaOMe (4 mg, 0.076 mmol) in MeOH (15 mL) and $L^2(PtCl)_2$ (23 mg, 0.015 mmol) in DCM (15 mL). In this instance, the crude material was purified by column chromatography on silica (DCM : MeOH, gradient to 5% MeOH) to give the desired product as a red solid (8.8 mg, 32%). ^{1}H NMR (600 MHz, CD_2Cl_2) δ_H = 9.86 (4 H, d, J 5.8, H^6), 7.61 (4 H, s, $H^{3'}$), 7.57 (2 H, d, J 2.4, H^d or $H^{d'}$), 7.51 (2 H, d, J 2.4, H^f or $H^{f'}$), 7.49 (4 H, s, H^3), 7.34 (2 H, d, J 2.4, H^d or $H^{d'}$), 7.33 (2 H, s, H^f or $H^{f'}$), 7.32 (4 H, d, J 2.2, H^5), 1.84 (6 H, s, H^{Me}), 1.74 (6 H, s, H^{Me}), 1.42 (18 H, s, $H^{t\text{-}Bu}$), 1.39 (18 H, s, $H^{t\text{-}Bu}$). ^{13}C NMR (151 MHz, CD_2Cl_2)[1] δ_C = 157.6 (C^6), 128.8 (C^f or $C^{f'}$), 126.5 ($C^{3'}$), 125.7 (C^5), 122.9 (C^d or $C^{d'}$), 119.8 (C^f or $C^{f'}$), 115.1 (C^3), 33.3 (C^{Me}), 32.3 (C^{Me}), 31.2 ($C^{t\text{-}Bu}$), 31.1 ($C^{t\text{-}Bu}$). HRMS (AP^+) *m/z* 1812.5026 $[M+H]^+$, calc. for $[C_{86}H_{72}F_{12}N_4O_2Pt_2]$ 1812.4858. λ_{max} / nm (ε / M^{-1} cm^{-1}): 270 (75900), 308 (43600), 415 (7590), 433 (7100), 494 (4800).

[1] HSQC was used to record ^{13}C peaks as the ^{13}C NMR spectrum was too weak owing to poor solubility of the compound.

L^3Pt_2(Xda)

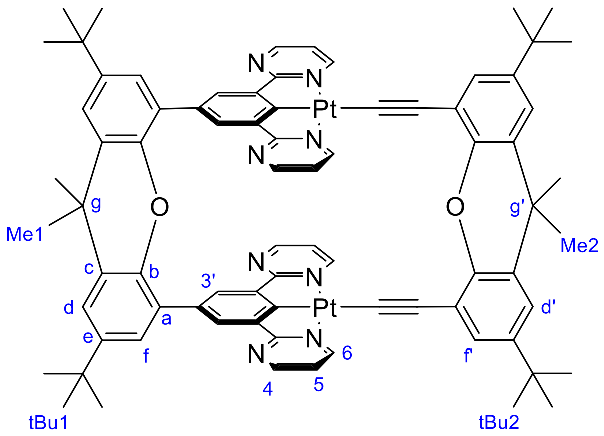

This compound was prepared similarly, from H_2Xda (6 mg, 0.015 mmol) and NaOMe (4 mg, 0.080 mmol) in MeOH (15 mL) and $L^3(Pt–Cl)_2$ (20 mg, 0.016 mmol) in DCM (15 mL). After washing the crude material with water, MeOH and Et_2O, it was purified by column chromatography on silica (DCM : MeOH, gradient to 5% MeOH) to give the product as a yellow solid (6.3 mg, 25%). ^{1}H NMR (600 MHz, CD_2Cl_2) δ_H = 9.70 (4 H, d, J 6.5, H^6), 8.44 (4 H, s, H^4), 7.75 (4 H, d, J 0.9, $H^{3'}$), 7.51 (2 H, s, H^d), 7.46 (2 H, s, $H^{d'}$), 7.31 (2 H, s, $H^{f'}$) 7.27 (2 H, s, H^f), 7.02 (4 H, t, J 5.2, H^5), 1.80 (6 H, s, H^{Me}), 1.70 (6 H, s, H^{Me}), 1.39 (18 H, d, J 0.8, $H^{t\text{-Bu}}$), 1.37 (18 H, s, $H^{t\text{-Bu}}$). ^{13}C NMR (151 MHz, CD_2Cl_2)[2] δ_C = 162.7 (C^6), 156.4 (C^4), 131.0 ($C^{3'}$), 128.9 (C^d), 125.5 (C^f), 122.2 ($C^{d'}$), 120.5 ($C^{f'}$), 118.6 (C^5), 33.2 (C^{Me}), 32.9 (C^{Me}), 31.6 ($C^{t\text{-Bu}}$), 31.5 ($C^{t\text{-Bu}}$). HRMS (AP^+) *m/z* = 1542.5154 [M^+], calc. for [$C_{78}H_{72}N_8O_2Pt_2$] 1542.5135. λ_{max} / nm (ε / M^{-1} cm^{-1}): 265 (78400), 308sh (25500), 396 (9250), 449 (9970).

[2] HSQC was used to assign ^{13}C peaks as the ^{13}C NMR spectrum was too weak due to insolubility of the compound.

HPLC Traces of $L^{2–3}Pt_2(Xda)$

SAMPLE INFORMATION

Sample Name:	RS278 C18XB	Acquired By:	Aileen
Sample Type:	Unknown	Sample Set Name	17April23_1
Vial:	1:E,1	Acq. Method Set:	Col3_5050MeCNgrad_MS
Injection #:	1	Processing Method	x
Injection Volume:	5.00 ul	Channel Name:	270.0nm
Run Time:	18.0 Minutes	Proc. Chnl. Descr.:	2998 PDA 270.0 nm (2998
Date Acquired:	17-Apr-23 5:40:44 PM BST		
Date Processed:	18-Apr-23 10:06:32 AM BST		

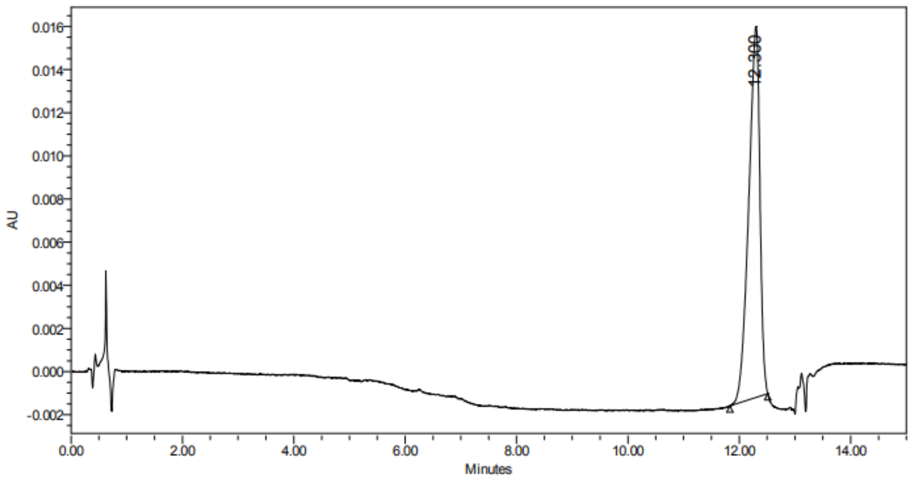

[redacted]				
[redacted]	12.300	238436	100.00	17185

Figure S1 *HPLC trace for $L^2Pt_2(Xda)$.*

SAMPLE INFORMATION			
Sample Name:	RS242 C18XB	Acquired By:	Aileen
Sample Type:	Unknown	Sample Set Name	17April23_1
Vial:	1:E,2	Acq. Method Set:	Col3_5050MeCNgrad_MS
Injection #:	2	Processing Method	x
Injection Volume:	5.00 ul	Channel Name:	270.0nm
Run Time:	18.0 Minutes	Proc. Chnl. Descr.:	2998 PDA 270.0 nm (2998
Date Acquired:	17-Apr-23 6:51:26 PM BST		
Date Processed:	18-Apr-23 10:05:53 AM BST		

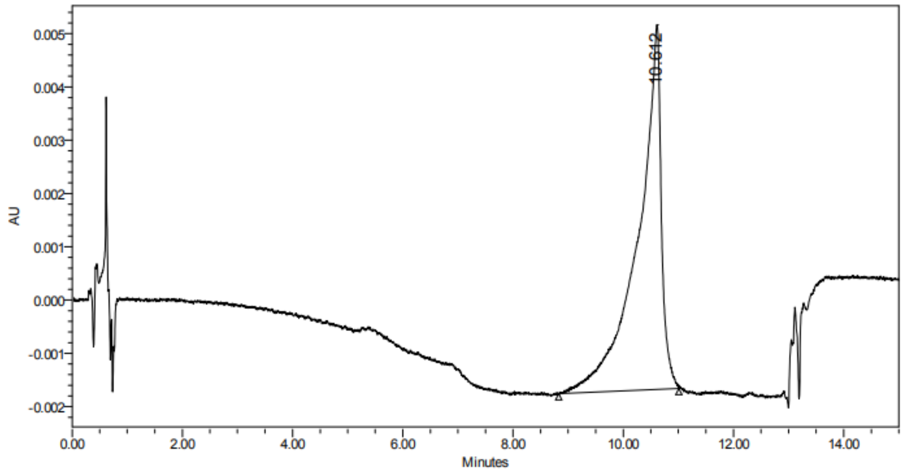

[redacted]				
[redacted]	10.612	199722	100.00	6841

Figure S2 *HPLC trace for $L^3Pt_2(Xda)$.*

^{1}H NMR Spectra

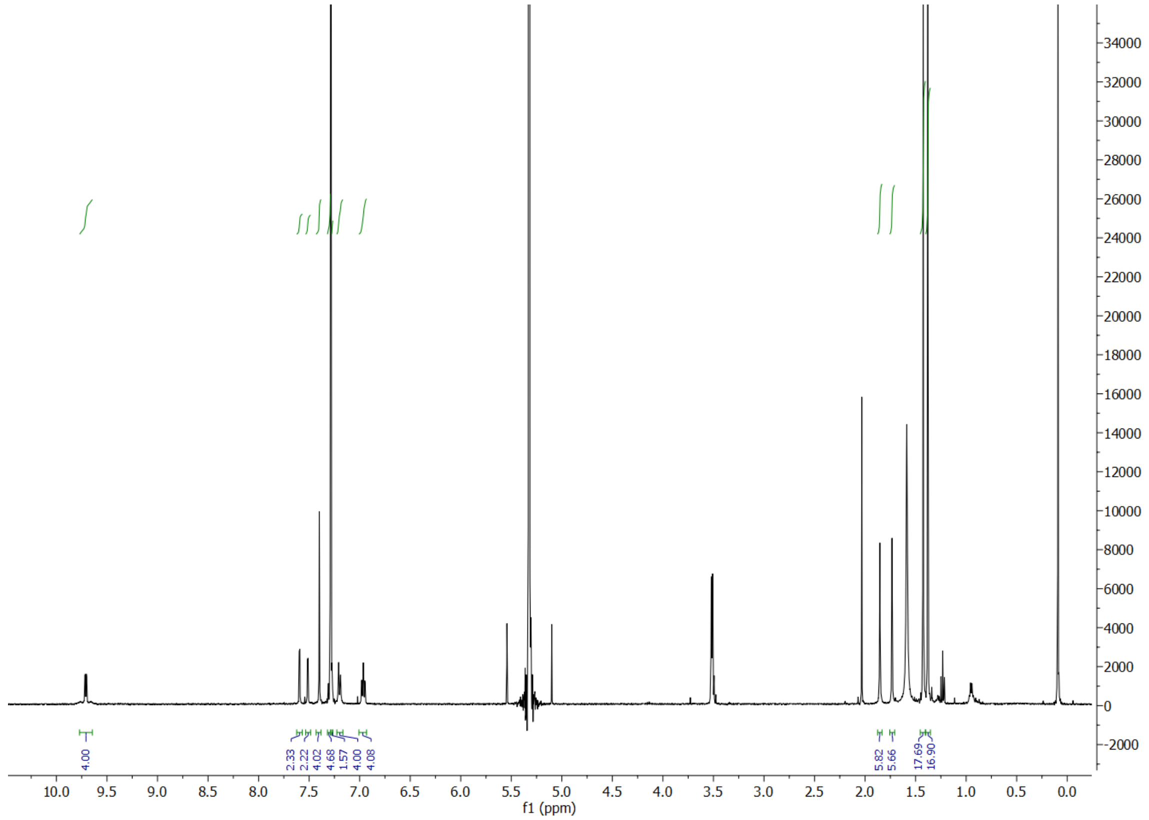

Figure S3 *^{1}H NMR spectrum of $L^{1}Pt_2(Xda)$ in $CDCl_3$ at 700 MHz.*

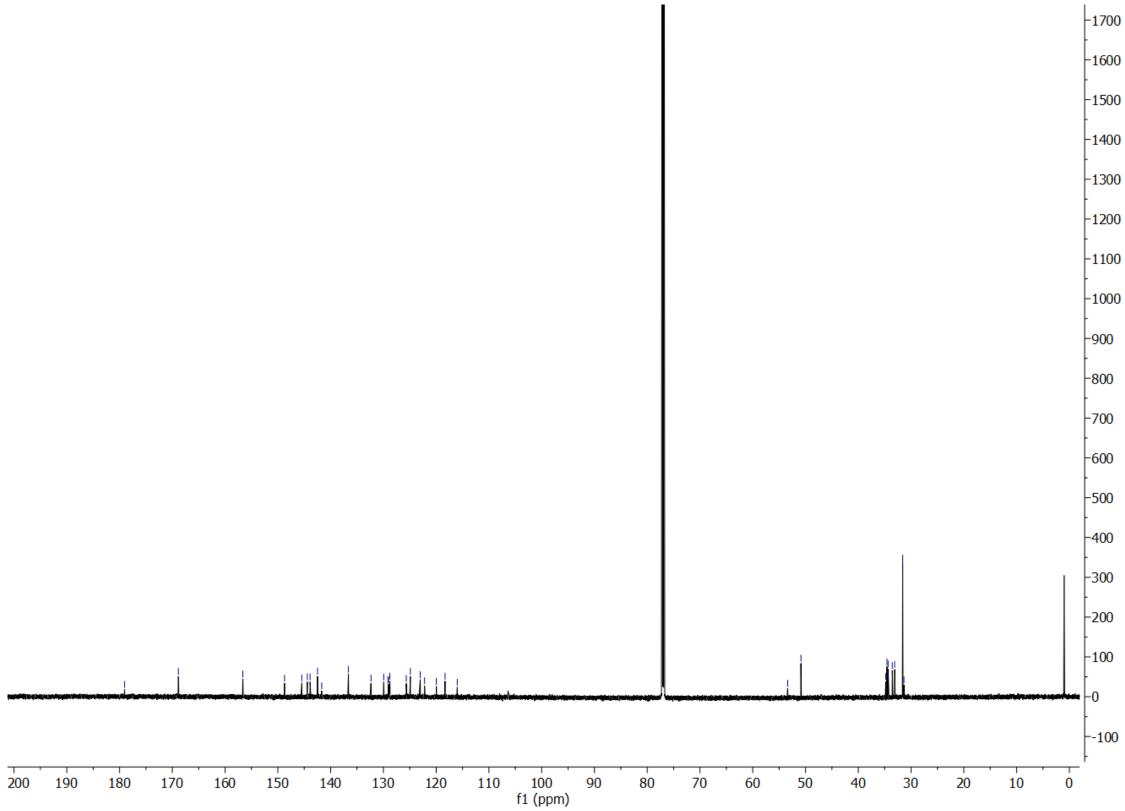

Figure S4 *^{13}C NMR spectrum of $L^{1}Pt_2(Xda)$ in $CDCl_3$ at 176 MHz.*

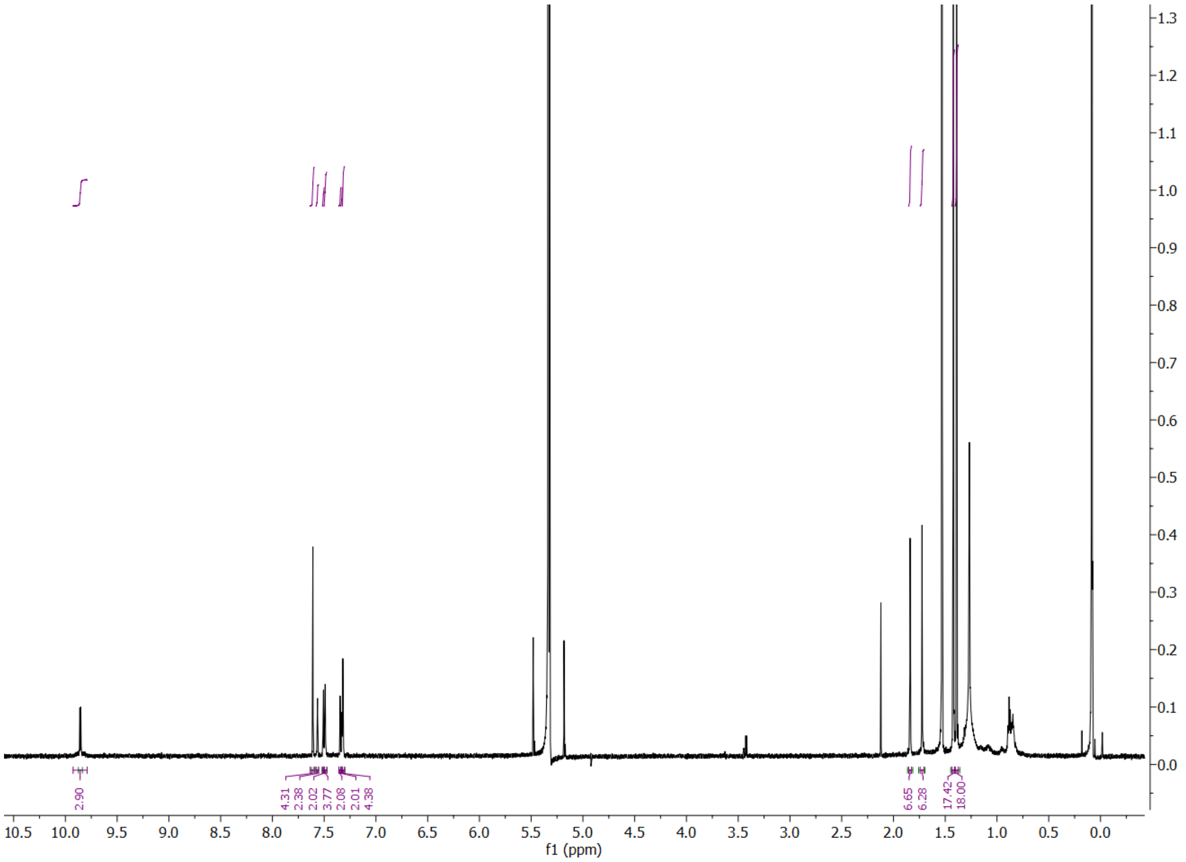

Figure S5 ^{1}*H NMR spectrum of $L^{2}Pt_2(Xda)$ in CD_2Cl_2 at 600 MHz.*

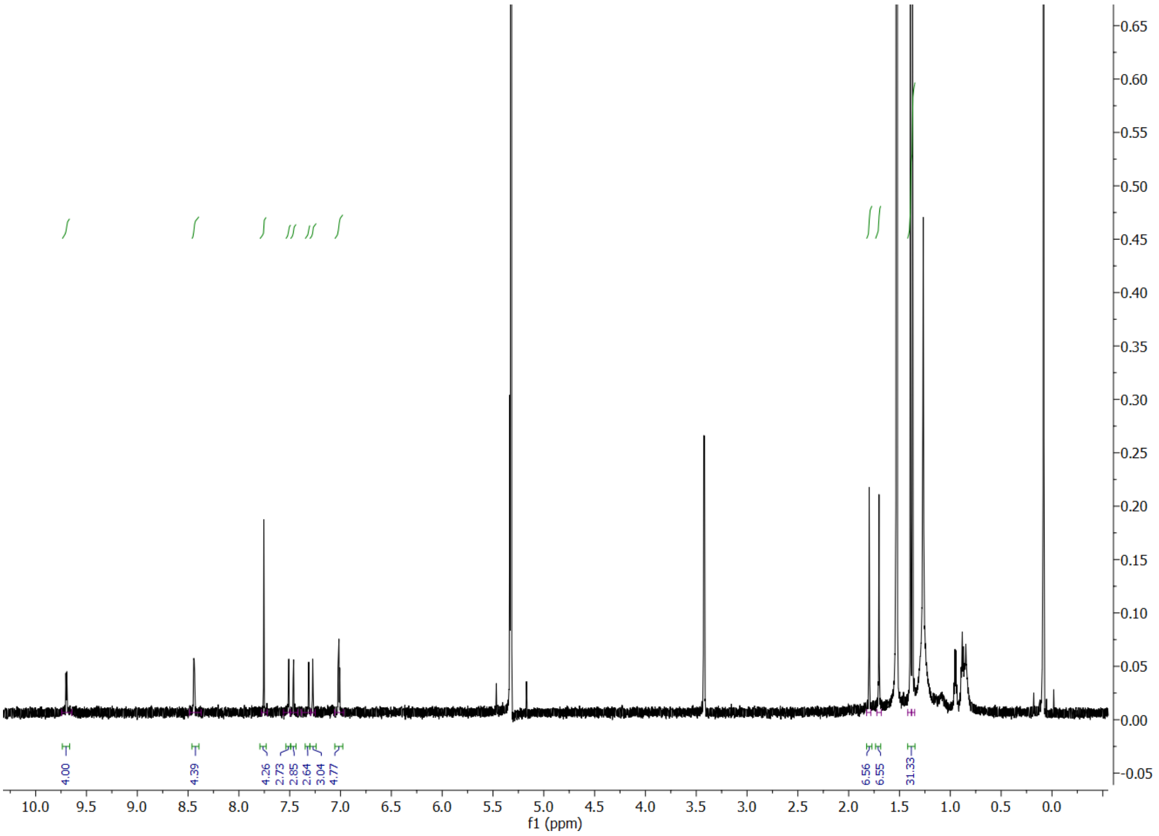

Figure S6 ^{1}*H NMR spectrum of $L^{3}Pt_2(Xda)$ in CD_2Cl_2 at 600 MHz.*

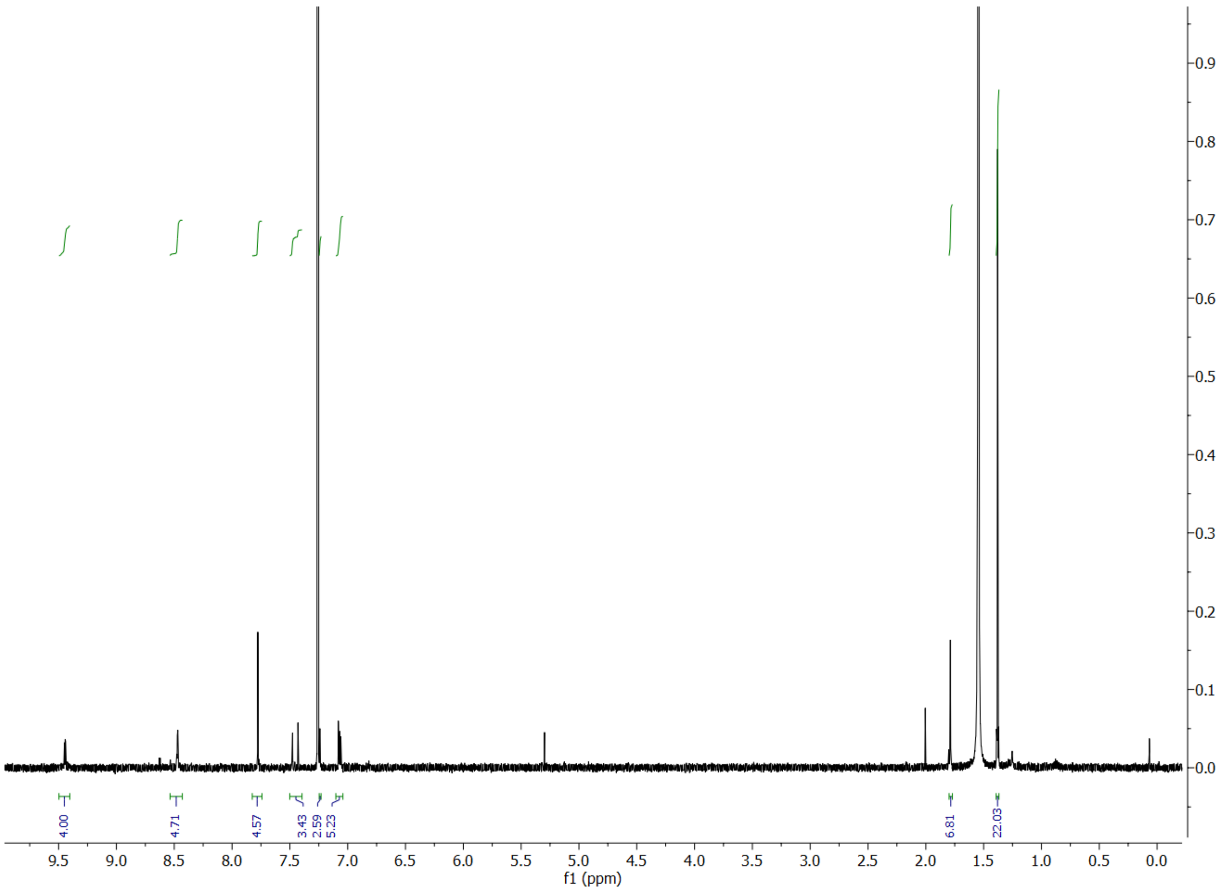

Figure S7 *^{1}H NMR spectrum of $L^3(Pt–Cl)_2$ in $CDCl_3$ at 600 MHz.*

Section 3 Crystallography

Table S1. Crystal data and structure refinement for L^1Pt_2(Xda)

Empirical formula	$C_{88}H_{82}Cl_{18}N_4O_2Pt_2$
Formula weight	2255.85
Temperature / K	120.0
Crystal system	monoclinic
Space group	$P2_1/n$
a / Å	20.6214(14)
b / Å	10.0682(7)
c / Å	23.5110(16)
α / °	90
β / °	94.520(2)
γ / °	90
Volume / Å^3	4866.2(6)
Z	2
ρ_{calc} g / cm^3	1.540
μ / mm^{-1}	3.412
F(000)	2232.0
Crystal size / mm^3	0.31 × 0.03 × 0.02
Radiation	Mo Kα (λ = 0.71073)
2Θ range for data collection / °	4.404 to 53.996
Index ranges	$-26 \leq h \leq 26$, $-12 \leq k \leq 12$, $-30 \leq l \leq 30$
Reflections collected	81882
Independent reflections	10614 [R_{int} = 0.1033, R_{sigma} = 0.0734]
Data/restraints/parameters	10614/165/444
Goodness-of-fit on F^2	1.032
Final R indexes [I >= 2σ (I)]	R_1 = 0.1029, wR_2 = 0.2790
Final R indexes [all data]	R_1 = 0.1361, wR_2 = 0.3025
Largest diff. peak / hole / e Å^{-3}	5.14 / -3.83

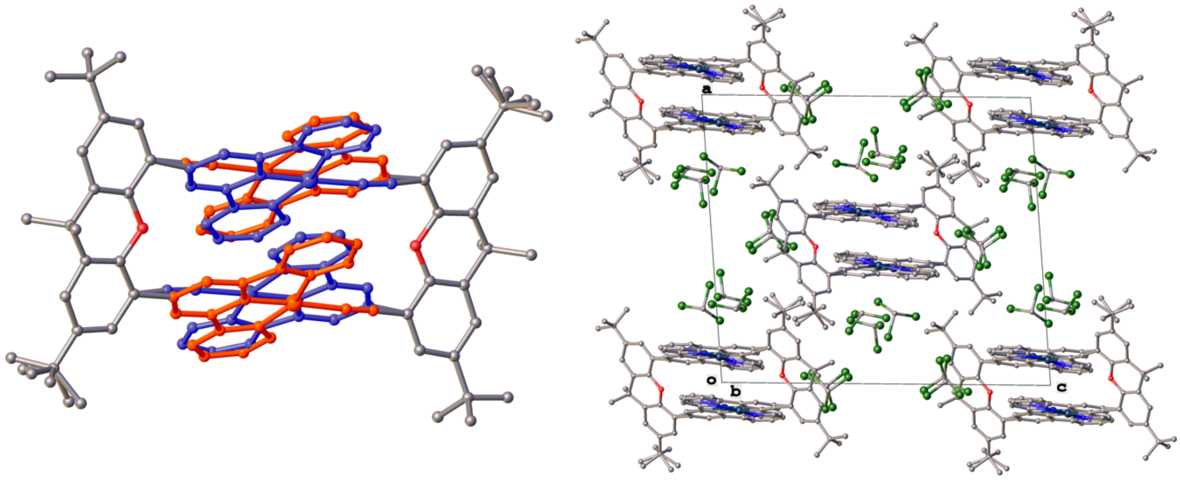

Figure S8 *Molecular and crystal structure of* $L^1Pt_2(Xda)$.

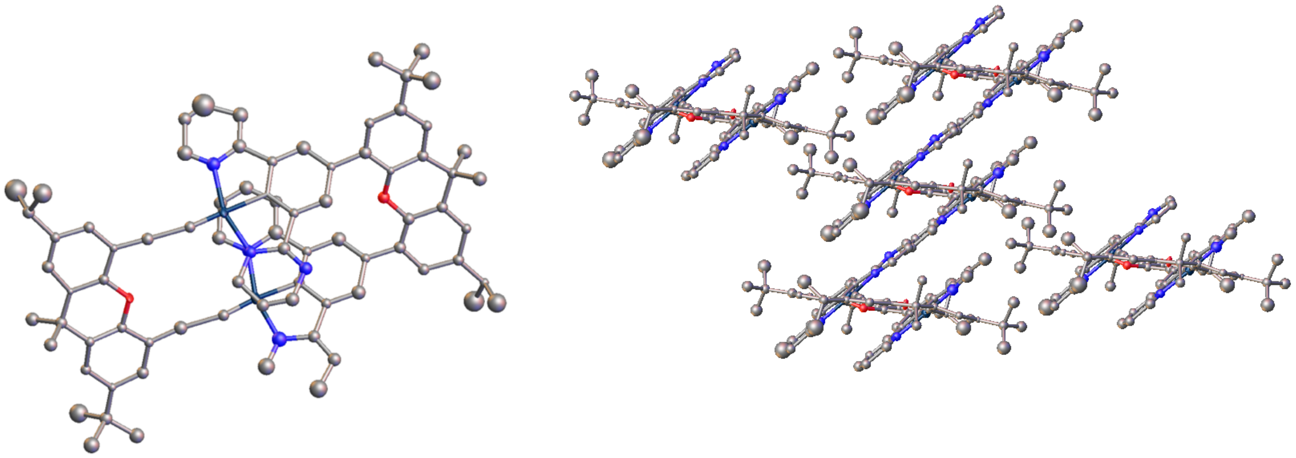

Figure S9 *Molecular and crystal structure of* $L^3Pt_2(Xda)$.

Section 4 Solution-state photophysics

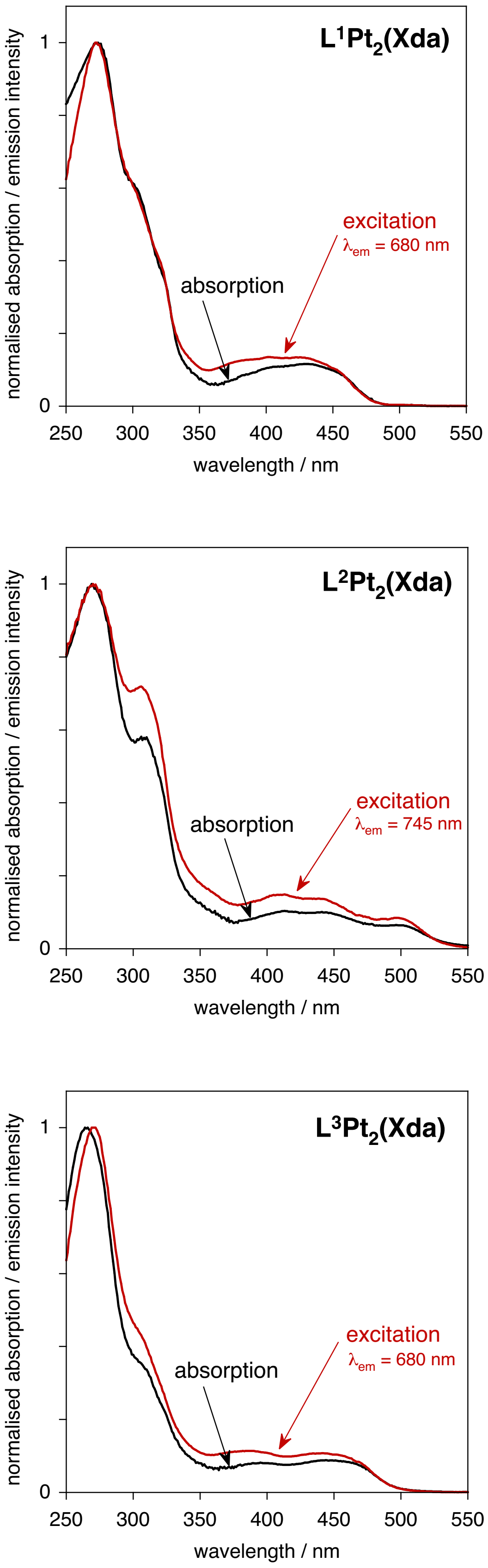

Figure S10 *Comparison of the absorption (black lines) and excitation spectra (red lines, λ_{em} as indicated) of* ***$L^{1-3}Pt_2$(Xda)*** *in CH_2Cl_2 solution at 295 K.*

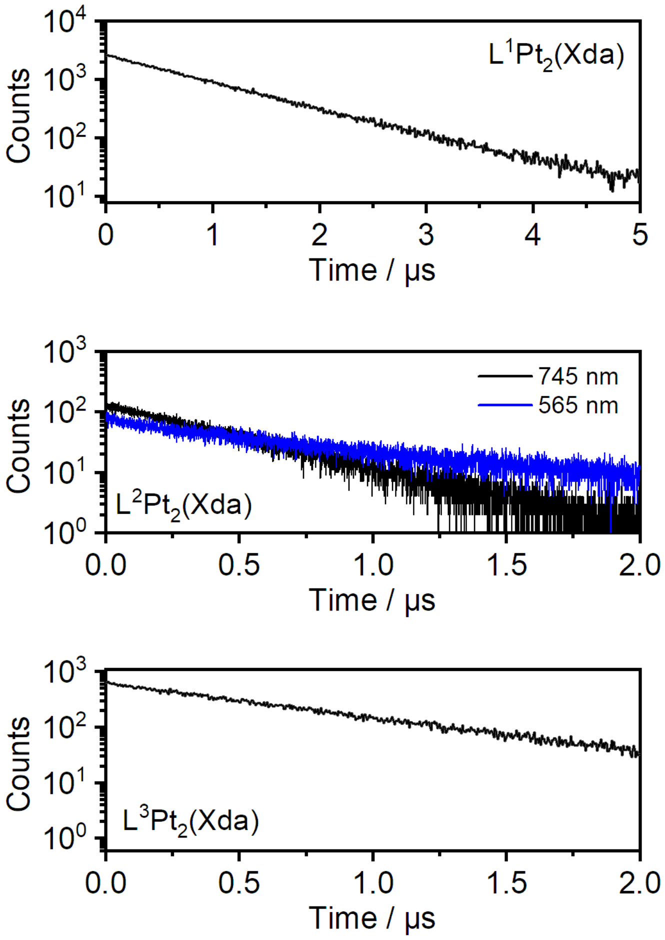

Figure S11 *Photoluminescence decay traces for* ***$L^{1-3}Pt_2(Xda)$*** *in CH_2Cl_2 solution (10^{-5} M). λ_{em} = 670 nm for* ***$L^1Pt_2(Xda)$****; 565 and 745 nm for* ***$L^2Pt_2(Xda)$****; 680 nm for* ***$L^3Pt_2(Xda)$****.*

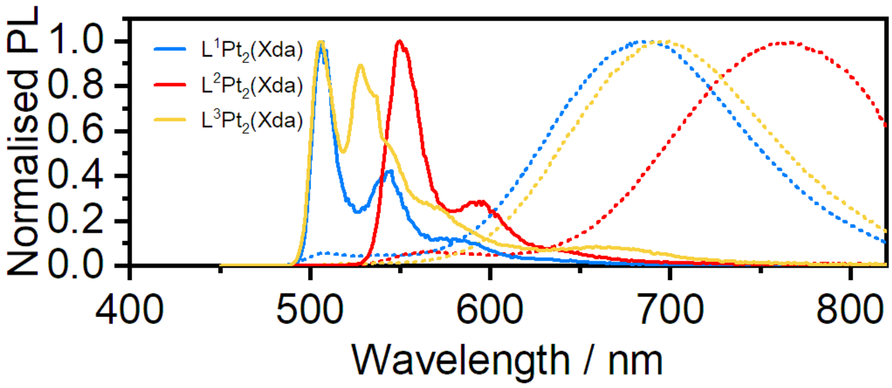

Figure S12 *Emission at 77 K (solid lines) compared to that at 295 K (dotted lines) of the macrocyclic compounds* ***$L^{1-3}Pt_2(Xda)$****.*

Section 5 Solid-state photophysics

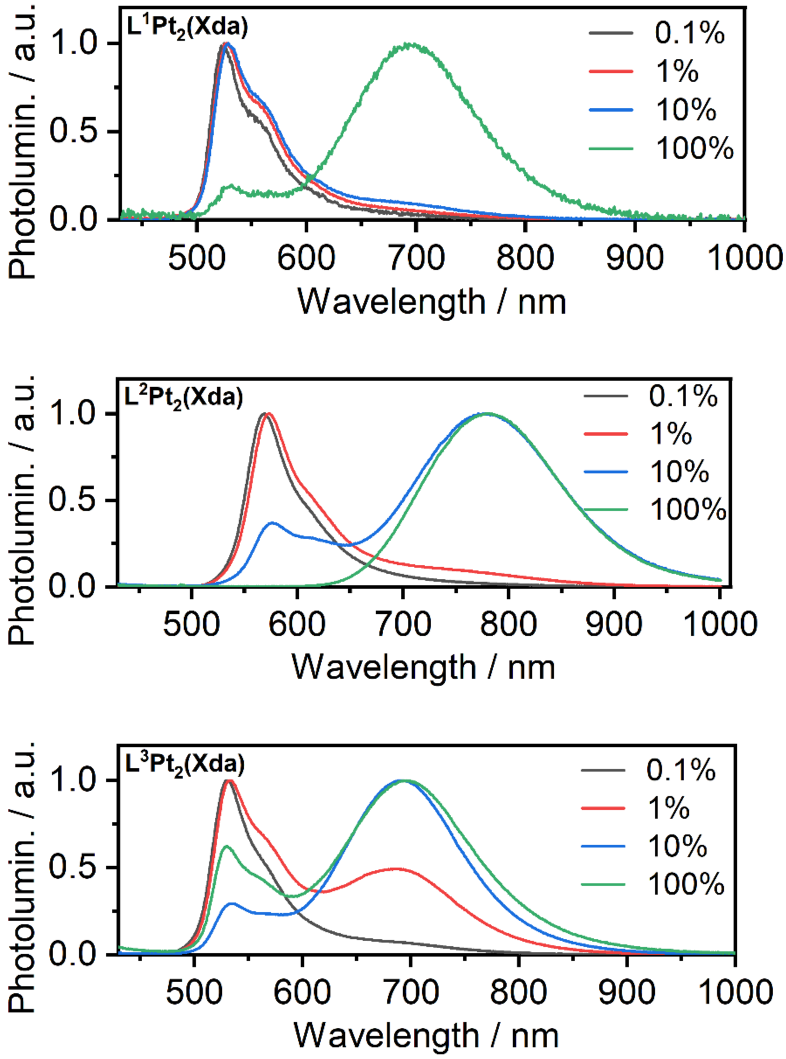

Figure S13 *Photoluminescence spectra of $L^{1-3}Pt_2(Xda)$ doped into polystyrene films at the loadings indicated (% by weight), at 295 K. (The spectra for $L^2Pt_2(Xda)$ were shown in the main text but are reproduced here to aid comparison).*

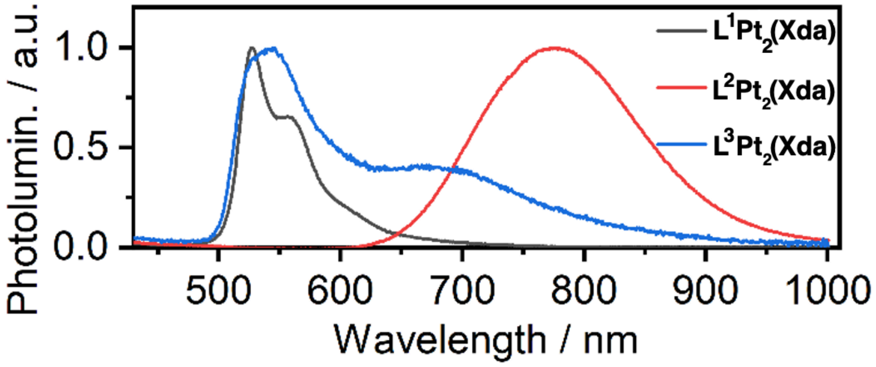

Figure S14 *Photoluminescence spectra of powdered "as-prepared" samples of $L^{1-3}Pt_2(Xda)$ at 295 K.*

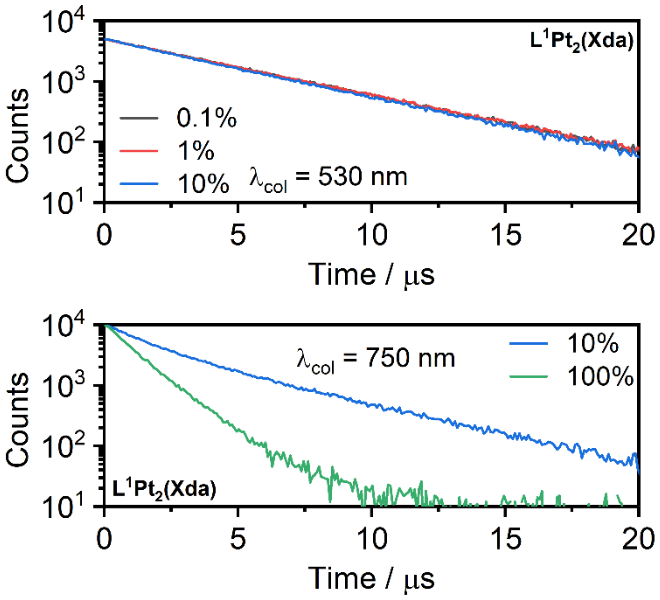

Figure S15 *Photoluminescence decay traces for $L^1Pt_2(Xda)$ in films.*

Top: λ_{em} = 530 nm. Bottom: λ_{em} = 750 nm.

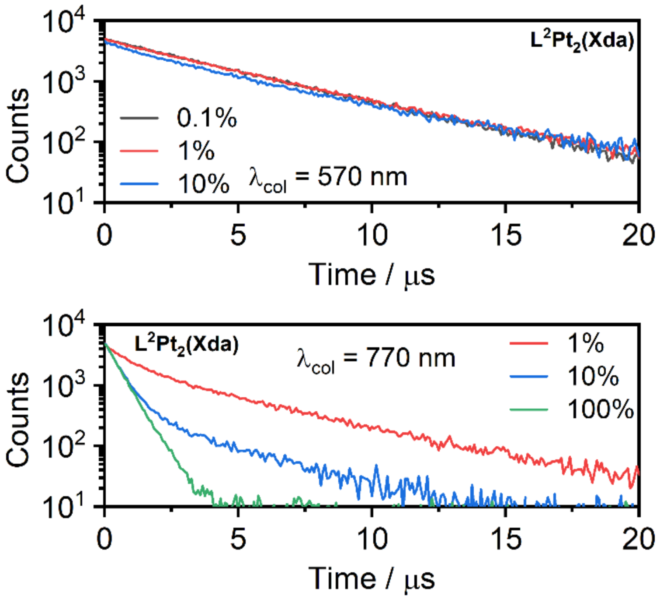

Figure S16 *Photoluminescence decay traces for* ***$L^2Pt_2(Xda)$*** *in films.*

Top: λ_{em} = 570 nm. Bottom: λ_{em} = 770 nm.

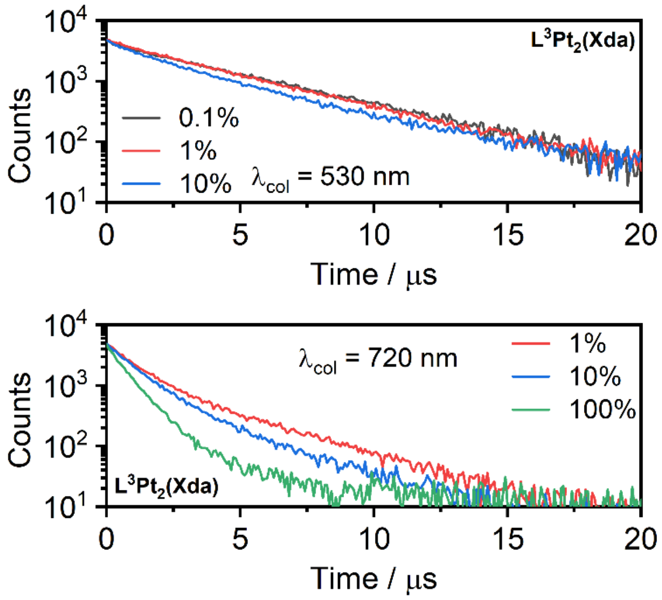

Figure S17 *Photoluminescence decay traces for* ***L^3Pt_2(Xda)*** *in films. Top: λ_{em} = 530 nm. Bottom: λ_{em} = 720 nm.*

Table S2. Photoluminescence decay times of **$L^{1\text{-}3}Pt_2$(Xda)** in films.

Complex	Concentration, %	λ_{col}, nm	τ, µs
**L^1Pt_2(Xda)**	0.1	530	4.7
		750	-
	1	530	4.7
		750	-
	10	530	4.5
		750	τ_1 = 4.5* (69%); τ_2 = 1.5 (31%)
	100	530	-
		750	1.1
**L^2Pt_2(Xda)**	0.1	570	4.2
		770	-
	1	570	4.3
		770	τ_1 = 4.5* (77%); τ_2 = 0.95 (23%)
	10	570	τ_1 = 5.0 (88%); τ_2 = 1.5 (12%)
		770	τ_1 = 0.53 (64%); τ_2 = 3.6 (36%)
	100	570	-
		770	0.57
**L^3Pt_2(Xda)**	0.1	530	4.3
		720	-
	1	530	4.2
		720	τ_1 = 3.4 (57%); τ_2 = 0.98 (43%)
	10	530	τ_1 = 5.3 (61%); τ_2 = 2.2 (39%)
		720	τ_1 = 1.1 (65%); τ_2 = 3.3 (35%)
	100	530	-
		720	τ_1 = 0.73 (81%); τ_2 = 2.8 (19%)

* indicates decay of the high-energy band [from excited states localized on a single Pt(*NCN*) unit].

Section 6 Calculations

Table S3. Characteristics of the two lowest singlet and triplet excited states at the S_0 geometry for **L^{1}(Pt–Cl)$_{2}$** and **L^{1}Pt$_{2}$(Xda)**

Complex	State	Energy, eV	Orbital character
**L^{1}(Pt–Cl)$_{2}$**	S_1	3.220	HOMO→LUMO (36%), HOMO-1→LUMO (20%), HOMO-1→LUMO+1 (17%), HOMO→LUMO+1 (10%)
	S_2	3.223	HOMO-1→LUMO (38%), HOMO→LUMO (19%), HOMO→LUMO+1 (15%), HOMO-1→LUMO+1 (11%)
	T_1	2.671	HOMO→LUMO (31%), HOMO-1→LUMO+1 (31%)
	T_2	2.686	HOMO-1→LUMO (33%), HOMO→LUMO+1 (32%)
**L^{1}Pt$_{2}$(Xda)**	S_1	3.025	HOMO→LUMO+1 (52%), HOMO-1→LUMO+1 (17%)
	S_2	3.044	HOMO-1→LUMO+1 (35%), HOMO→LUMO (29%)
	T_1	2.632	HOMO-1→LUMO+1 (14%), HOMO→LUMO+1 (11%), HOMO-1→LUMO (10%)
	T_2	2.642	HOMO→LUMO+1 (18%), HOMO-1→LUMO+1 (12%), HOMO-2→LUMO+1 (10%)

Table S4. Characteristics of the S_1 excited state at the T_1 geometry for all complexes discussed.

Complex	State	Energy, eV	Orbital character
**L^{1}(Pt–Cl)$_{2}$**	S_1	2.103	HOMO→LUMO (80%), HOMO-1→LUMO (10%)
**L^{1}Pt$_{2}$(Xda)**	S_1	2.367	HOMO→LUMO (60%), HOMO-1→LUMO (21%)
**L^{2}(Pt–Cl)$_{2}$**	S_1	1.960	HOMO→LUMO (87%), HOMO-1→LUMO (9%)
**L^{2}Pt$_{2}$(Xda)**	S_1	2.259	HOMO→LUMO (67%), HOMO-2→LUMO (23%)
**L^{3}(Pt–Cl)$_{2}$**	S_1	2.079	HOMO→LUMO (86%), HOMO-1→LUMO (9%)
**L^{3}Pt$_{2}$(Xda)**	S_1	2.379	HOMO→LUMO (62%), HOMO-2→LUMO (17%), HOMO-1→LUMO (11%)

Table S5. Characteristics of the T_1 excited state at the T_1 geometry for all complexes discussed.

Complex	State	Energy, eV	Orbital character
$\mathbf{L^1(Pt–Cl)_2}$	T_1	1.920	HOMO→LUMO (70%), HOMO-1→LUMO (12%)
$\mathbf{L^1Pt_2(Xda)}$		2.148	HOMO→LUMO (53%), HOMO-1→LUMO (24%)
$\mathbf{L^2(Pt–Cl)_2}$		1.788	HOMO→LUMO (80%), HOMO-1→LUMO (12%)
$\mathbf{L^2Pt_2(Xda)}$		2.089	HOMO→LUMO (56%), HOMO-2→LUMO (29%)
$\mathbf{L^3(Pt–Cl)_2}$		1.903	HOMO→LUMO (78%), HOMO-1→LUMO (12%)
$\mathbf{L^3Pt_2(Xda)}$		2.177	HOMO→LUMO (51%), HOMO-2→LUMO (17%), HOMO-1→LUMO (13%)

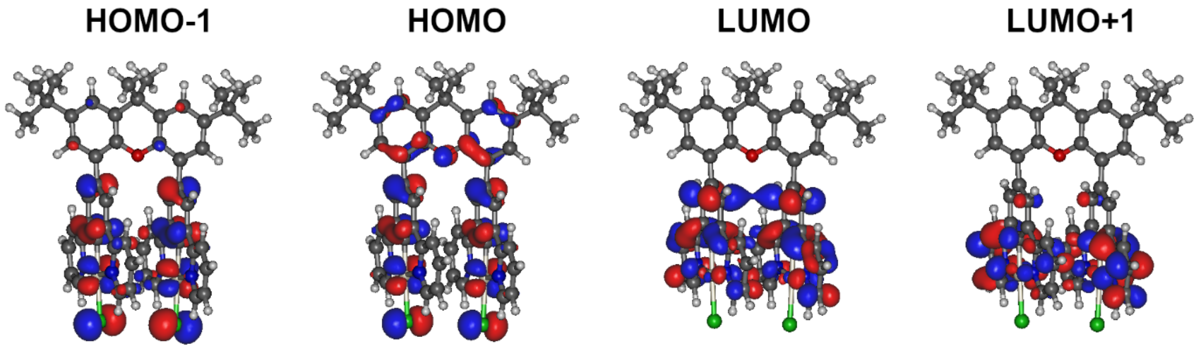

Figure S18 *Isosurfaces of molecular orbitals relevant to S_1 and T_1 at the S_0 geometry of* $\mathbf{L^1(Pt–Cl)_2}$.

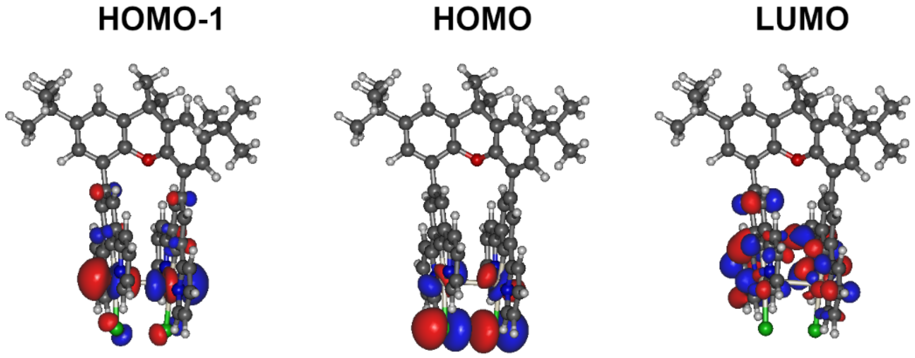

Figure S19 *Isosurfaces of molecular orbitals relevant to S_1 and T_1 at the T_1 geometry of* $\mathbf{L^1(Pt–Cl)_2}$.

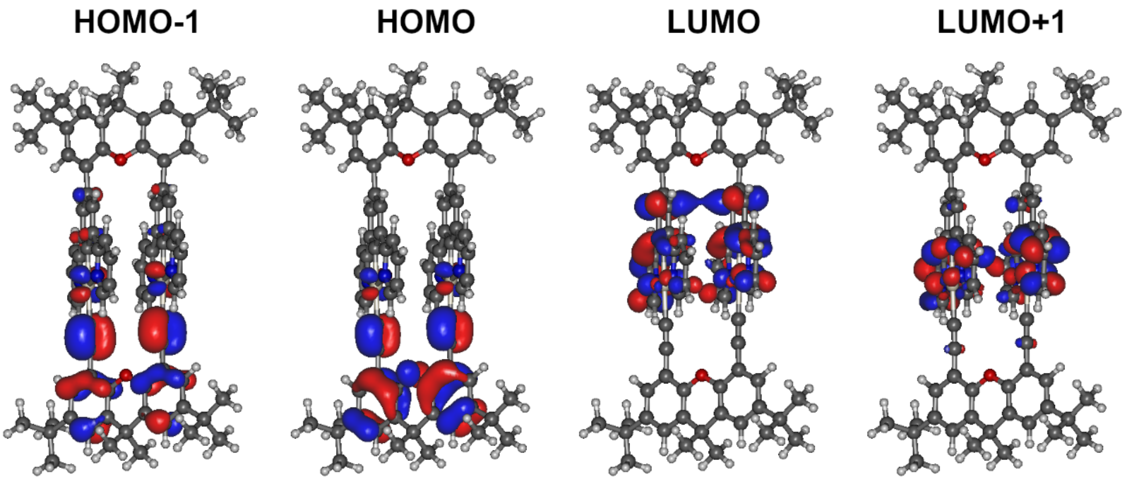

Figure S20 *Isosurfaces of molecular orbitals relevant to S_1 and T_1 at the S_0 geometry of* ***L^1Pt_2(Xda)***.

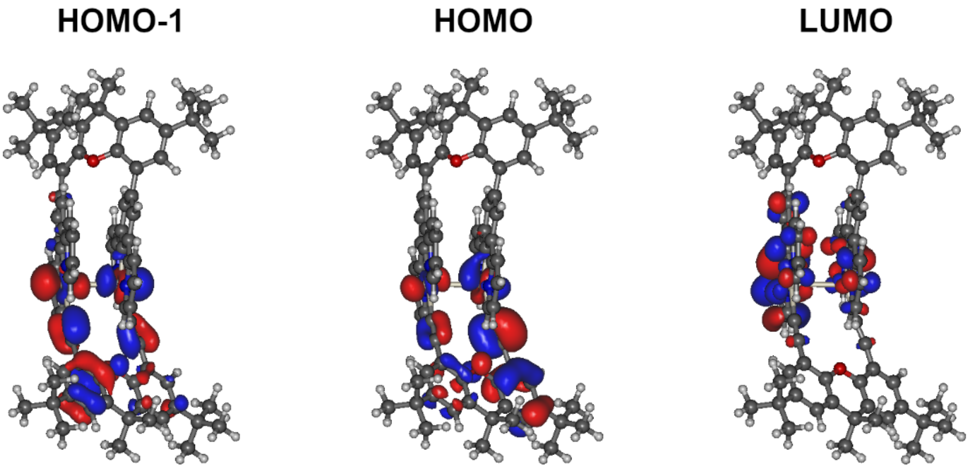

Figure S21 *Isosurfaces of molecular orbitals relevant to S_1 and T_1 at the T_1 geometry of* ***L^1Pt_2(Xda)***.

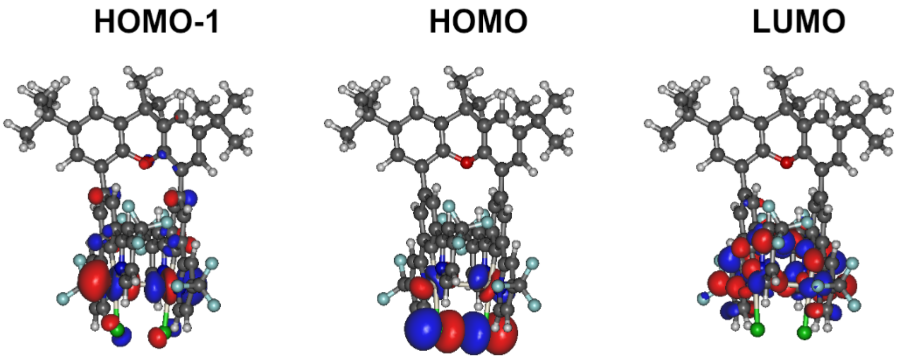

Figure S22 *Isosurfaces of molecular orbitals relevant to S_1 and T_1 at the T_1 geometry of* ***L^2(Pt–Cl)$_2$***.

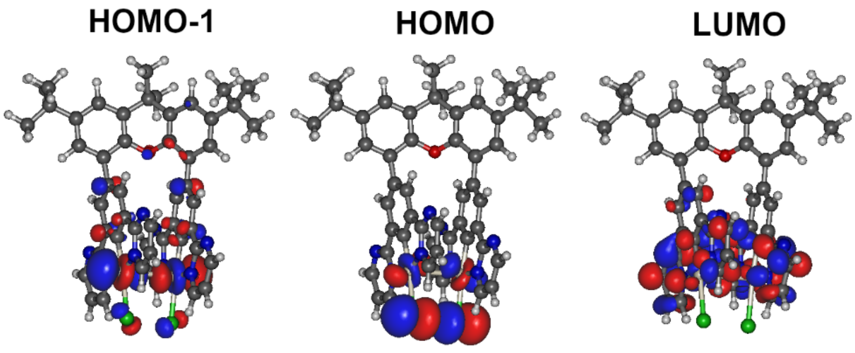

Figure S23 *Isosurfaces of molecular orbitals relevant to S_1 and T_1 at the T_1 geometry of* ***L^3(Pt–Cl)$_2$***.

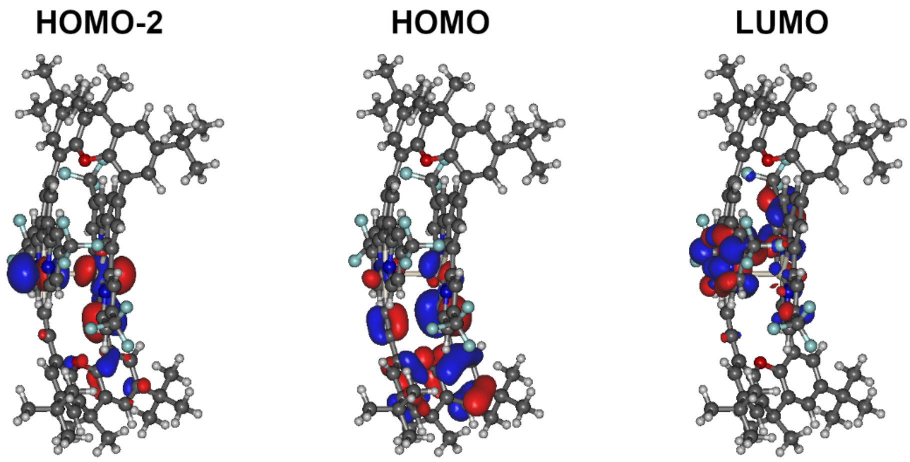

Figure S24 *Isosurfaces of molecular orbitals relevant to S_1 and T_1 at the T_1 geometry of* ***L^2Pt_2(Xda)***.

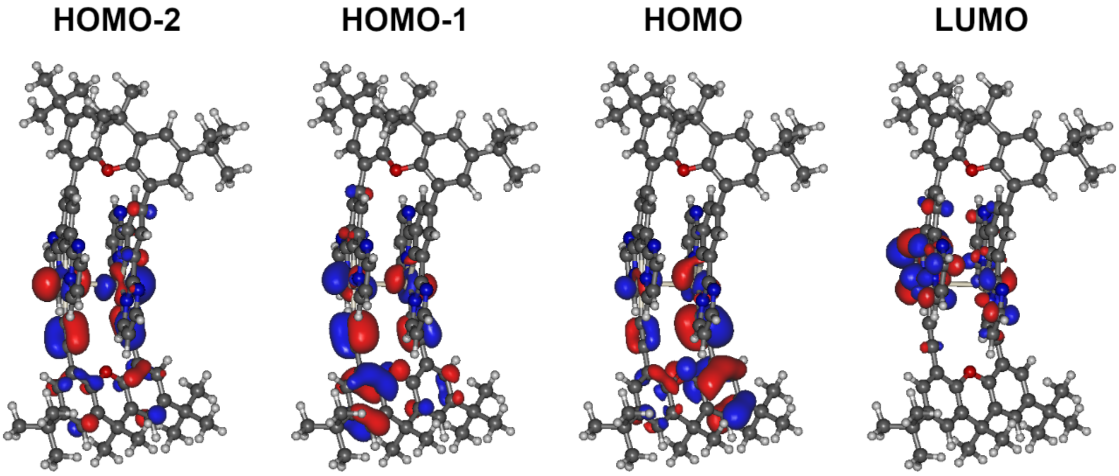

Figure S25 *Isosurfaces of molecular orbitals relevant to S_1 and T_1 at the T_1 geometry of* ***L^3Pt_2(Xda)***.

References

1 F. Neese, *WIREs Comput. Mol. Sci.*, **2012**, *2*, 73–78.

2 S. Lehtola, C. Steigemann, M. J. T. Oliveira, M. A. L. Marques, *SoftwareX*, **2018**, *7*, 1–5.

3 F. Neese, *WIREs Comput. Mol. Sci.*, **2022**, 12:e1606.

4 A.-R. Allouche, *J. Comput. Chem.*, **2011**, *32*, 174–182.

5 A. D. Becke, *Phys. Rev. A*, **1988**, *38*, 3098–3100.

6 F. Weigend, R. Ahlrichs, *Phys. Chem. Chem. Phys.*, **2005**, *7*, 3297.

7 T. Yanai, D. P. Tew, N. C. Handy, *Chem. Phys. Lett.*, **2004**, *393*, 51–57.

8 F. Neese, F. Wennmohs, A. Hansen, U. Becker, *Chem. Phys.*, **2009**, *356*, 98–109.

9 R. Izsák, F. Neese, *J. Chem. Phys.*, **2011**, *135*, 144105.

10 F. Weigend, *Phys. Chem. Chem. Phys.*, **2006**, *8*, 1057.

11 S. Grimme, S. Ehrlich, L. Goerigk, *J. Comput. Chem.*, **2011**, *32*, 1456–1465.

12 S. Grimme, J. Antony, S. Ehrlich, H. Krieg, *J. Chem. Phys.*, **2010**, *132*, 154104.

13 S. Develay, J. A. G. Williams, *Dalton Trans.*, **2008**, 4562.

14 P. Pander, M. T. Walden, R. J. Salthouse, A. Sil, D. S. Yufit, F. B. Dias, J. A. G. Williams, *J. Mater. Chem. C*, **2023**, *11*, 15335–15346.

15 R. Muñoz-Rodríguez, E. Buñuel, J. A. Williams, D. J. Cárdenas, *Chem. Commun.*, **2012**, *48*, 5980.
